# Structural vibration monitoring with diffractive optical processors

**DOI:** 10.1126/sciadv.aea1712

**Published:** 2026-03-04

**Authors:** Yuntian Wang, Zafer Yilmaz, Yuhang Li, Edward Liu, Eric Ahlberg, Farid Ghahari, Ertugrul Taciroglu, Aydogan Ozcan

**Affiliations:** ^1^ Electrical and Computer Engineering Department, University of California, Los Angeles, Los Angeles, CA 90095, USA.; ^2^ Bioengineering Department, University of California, Los Angeles, Los Angeles, CA 90095, USA.; ^3^ California NanoSystems Institute (CNSI), University of California, Los Angeles, Los Angeles, CA 90095, USA.; ^4^ Civil and Environmental Engineering Department, University of California, Los Angeles, Los Angeles, CA 90095, USA.; ^5^ California Geological Survey, California Department of Conservation, Sacramento, CA 95814, USA.

## Abstract

Structural health monitoring (SHM) is vital for maintaining the safety and longevity of civil infrastructure, yet current solutions remain constrained by cost, power consumption, scalability, and the complexity of data processing. Here, we present a diffractive vibration monitoring system, integrating a jointly optimized diffractive layer with a shallow neural network-based backend to remotely extract three-dimensional (3D) structural vibration spectra, offering a low-power, cost-effective, and scalable solution. Unlike prior diffractive processors designed primarily for static image classification or reconstruction tasks, this framework establishes a dynamic computational sensing modality where the optical front-end is co-optimized to encode time-varying mechanical vibrations into distinct spatiotemporal optical patterns. This architecture eliminates the need for dense sensor arrays or extensive data acquisition; instead, it uses a spatially optimized passive diffractive layer that encodes 3D structural displacements into modulated light, captured by a minimal number of detectors and decoded in real time by shallow and low-power neural networks to reconstruct the 3D displacement spectra of structures. The diffractive system’s efficacy was demonstrated both numerically and experimentally using millimeter-wave illumination on a laboratory-scale building model with a programmable shake table. Our system achieves more than an order-of-magnitude improvement in accuracy over conventional optics or separately trained modules, establishing a foundation for high-throughput 3D monitoring of structures. Beyond SHM, the 3D vibration monitoring capabilities of this cost-effective and data-efficient framework establish a distinct computational sensing modality with potential applications in disaster resilience, aerospace diagnostics, and autonomous navigation—where energy efficiency, low latency, and high-throughput are critical.

## Teaser

A diffractive optical processor is designed to remotely monitor 3D structural vibrations with high accuracy and low power.

## INTRODUCTION

Ensuring the safety and longevity of civil infrastructure, such as buildings, bridges, and dams, is paramount for societal well-being and economic stability (*1*–*4*). Structural health monitoring (SHM) systems play a critical role in this endeavor by providing methods to assess the conditions of structures, detect damage, and predict the remaining service life, particularly after exposure to natural hazards like earthquakes (*5*, *6*). Traditional SHM systems often rely on visual inspections, which suffer from notable drawbacks: They require highly skilled personnel, can be time-consuming, costly, dangerous, and subjective, and may not always be feasible for inaccessible parts of a structure or large-scale assessments (*7*). Alternative approaches include nondestructive testing (NDT) (*8*–*11*) and vibration-based SHM (*12*–*15*). For example, vibration-based methods use sensor networks (e.g., accelerometers and strain gauges) to record structural responses to excitations. The analysis of these data, often through system identification techniques (*16*–*36*), aims to extract modal parameters (frequencies, damping ratios, and mode shapes) whose changes can indicate damage. Although powerful, conventional sensor networks can be expensive to install and maintain, require considerable power, and generate large datasets demanding complex digital signal processing. Furthermore, achieving high spatial resolution for accurate damage localization often necessitates a dense and costly sensor deployment. Recent advancements like digital twins (*37*–*42*) leverage these methods but still depend on the quality and density of the underlying sensor data. More recent sensing technologies, including fiber optics (*43*), laser Doppler vibrometry (*44*, *45*), and vision-based systems (*46*, *47*), offer improvements but still face challenges in their relative costs, deployment complexity, or sensitivity.

Here, we present a structural vibration monitoring approach integrating diffractive processor-based encoders with shallow neural network-based decoders to accurately and rapidly reconstruct the three-dimensional (3D) oscillation amplitudes and frequencies of a structure under test. This approach extends the capabilities of diffractive optical processors from static inference to dynamic state estimation, using an optimized, passive physical encoder to process continuous-time signals generated by 3D mechanical motion or vibrations. In this architecture, an optimized encoder surface with wavelength scale diffractive features is attached to the structure of interest; as it oscillates at various frequencies, the corresponding movement of the passive diffractive layer modulates the reflected wavefront. This modulated light is captured by a few detectors, generating time-series signals that encode the 3D vibration spectra of the test structure. Shallow and low-power neural networks, co-optimized with the diffractive layer design, rapidly decode the detector signals to quantify the 3D displacement spectra of the structure. This diffractive 3D vibration monitoring approach offers the potential for a low-power, cost-effective, and scalable solution for parallel monitoring of structures. The passive nature of the structurally optimized diffractive layer reduces power requirements, whereas the computational capabilities of the deep learning–based backend enable robust decoding of vibration spectra from sparse detector data, also eliminating the acquisition and storage of large amounts of data. This co-optimization strategy ensures that the passive diffractive layer is specifically tailored to encode the structural displacements in a way that is optimally decodable by a shallow and low-power backend network, maximizing sensitivity and accuracy while also reducing complexity and digital data burden.

**Fig. 1.**
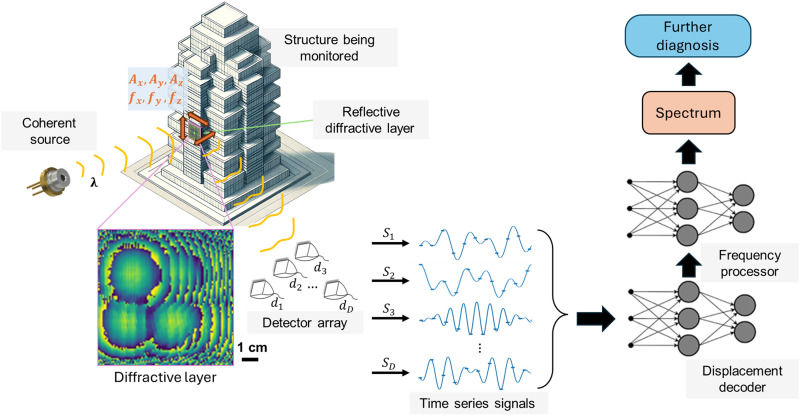
Schematic of a diffractive optical processor designed for high-throughput structural vibration monitoring with a shallow electronic neural network-based decoding. The diffractive optical processor, optimized using deep learning, is attached at various locations on a target structure, where it encodes local oscillatory motion into wavelength-dependent structurally optimized diffraction patterns. A few detectors capture the reflected light, which varies in signal strength based on the displacement amplitudes and frequencies at each point that is being monitored. The resulting time series signals are rapidly decoded by a shallow neural network backend (including a displacement decoder and a frequency processor) to simultaneously extract 3D oscillation features of the structure under test. This integrated system can enable cost-effective, low-power structural health diagnostics powered by deep learning–designed diffractive processors.

Our results and analysis demonstrate the validation of this diffractive vibration monitoring concept (Fig. 1), revealing its accuracy in extracting the vibration spectra of different structures. We provide its experimental proof of concept using millimeter-wave illumination and a 3D-printed diffractive layer mounted on a laboratory-scale building model subjected to 1D and 2D dynamic excitations. We show that the jointly optimized diffractive system significantly outperforms other configurations using conventional optical elements, validating the efficacy of the jointly optimized design for high-fidelity vibration monitoring of structures with more than an order of magnitude improvement (detailed in the Results section) in accuracy.

The approach presented here marks a fundamental departure from conventional digital sensing paradigms by shifting a portion of the computational burden into the physical domain. This is achieved through the codesign and joint optimization of a passive diffractive encoder and a shallow, low-power neural network decoder. Unlike traditional sensor networks that digitize raw physical signals for subsequent extensive digital processing, our system leverages the diffractive layer as an optimized optical processor that intelligently preencodes complex, multidimensional structural oscillation information directly into modulated optical signals. By exploiting the mechanical-optical coupling to encode temporal dynamics, the diffractive layer effectively functions as an optimized signal processor in the physical domain, transforming continuous mechanical vibrations into intensity variations specifically tailored for a jointly optimized temporal decoder. This physical-digital cointegration provides a new foundation for real-time, energy-efficient computational sensing systems that are not limited to civil infrastructure. By reframing the problem of structural vibration monitoring as an optical inference task, our work introduces a compact, scalable, and cost-effective platform with broad relevance to distributed sensing in resource-limited settings, from smart cities and disaster prevention to aerospace diagnostics and autonomous navigation. These results represent not just a new tool for SHM, but a novel modality in physical computation and signal encoding, capable of transforming how we remotely sense and interpret dynamic physical systems. Furthermore, this system exemplifies the principles of edge AI in the physical domain, where hardware is not merely a conduit for digital inference but an active and co-optimized participant in the computational process itself. Such advances could lay the groundwork for future systems that seamlessly integrate sensing, encoding, and inference into unified, compact optical layers—paving the way for scalable networks of intelligent sensors deployed at an unprecedented scale across various scientific and engineering disciplines.

## RESULTS

### Diffractive systems for monitoring 3D structural vibrations

**Fig. 2.**
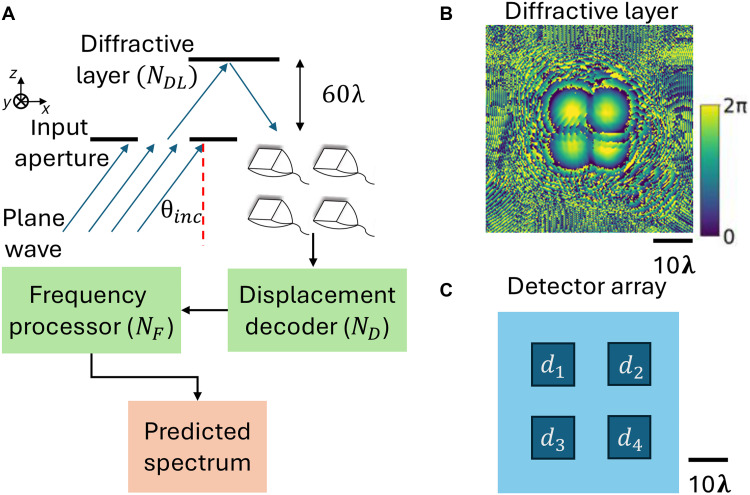
Configuration and components of a diffractive vibration monitoring system. (**A**) System configuration: An input wave (incident at $\theta_{\mathrm{inc}}$) reflects off of an optimized diffractive layer, which is attached to an oscillating structure. The resulting optical signal (in reflection) is captured by a detector array and rapidly processed by a displacement decoder and a frequency processor, which measure the displacement and the 3D oscillation spectrum of the structure under test. $N_{\mathrm{DL}}$, $N_{D}$, and $N_{F}$ are the number of trainable parameters in the optimized diffractive layer, displacement decoder network, and frequency processor network, respectively. (**B**) Optimized phase modulation pattern of the trained surface of the diffractive vibration monitoring system. The diffractive processor phase profile was jointly trained with a displacement decoder that has $N_{D}=6.39k$ trainable parameters. (**C**) Detector array layout with four single-pixel detectors.

We designed a diffractive system capable of measuring and extracting 3D structural oscillations using an optimized reflective layer. The system configuration is depicted in Fig. 2A. Input wave, incident at an oblique angle relative to the normal, propagates through free space onto the reflective diffractive layer (shown in Fig. 2B). This reflective diffractive surface, affixed to the target structure under test, consists of a 200 by 200 array of trainable, phase-only diffractive features, each approximately λ/2 in lateral size, where λ is the illumination wavelength. Mechanically attached to the structure, this passive diffractive layer follows the structure’s displacements, modeled as a linear combination of various harmonics in *x*, *y*, and *z* directions with randomly generated amplitudes and phases (see Methods for details). The reflected wave that is spatiotemporally modulated by the optimized diffractive layer propagates to the output plane and is sampled by four detectors at 50 Hz. The detected time signals serve as an encoded input to a shallow displacement decoder network that rapidly estimates the 3D structural displacement time series (see the section “Network structure for the digital backend” in the Methods for architectural details). The detector array contains four single-pixel detectors arranged uniformly, with a center-to-center spacing of 16λ (Fig. 2C). The detected time signals serve as an encoded input to a shallow displacement decoder network that estimates the 3D structural displacement time series, i.e., *x*(*t*), *y*(*t*), and *z*(*t*). A subsequent frequency processor network then analyzes the displacement data to extract the 3D oscillation spectra, covering a predetermined range of 9 to 11 Hz (selected, without loss of generality, as the target band of interest) in *x*, *y*, and *z* directions (Fig. 2A). This selected spectral range aligns with the fundamental frequencies of the experimental model. This selection is further justified by its applicability to civil infrastructure; specifically, for low-rise buildings, the fundamental mode (typically associated with the highest modal participation) often falls within this range, although the exact frequency may vary depending on the structural type. The ease of redesigning the diffractive layer allows for practical adjustments to accommodate different configurations.

**Fig. 3.**
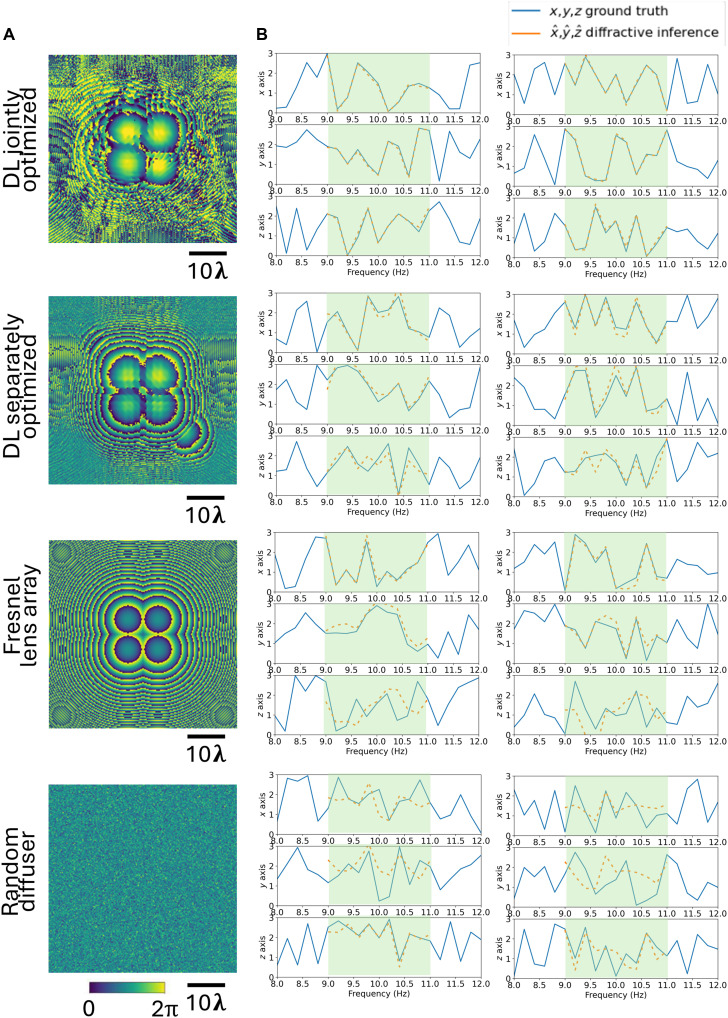
Comparison of 3D oscillation spectra inference performance across different optical configurations; $N_{D}=2.98k$. (**A**) Phase modulation patterns of a jointly trained diffractive layer, a separately trained diffractive layer, a Fresnel lens array, and a random phase diffuser, displayed from top to bottom, respectively. (**B**) Ground truth 3D oscillation spectra (two examples in each direction) and the diffractive inference results for each configuration. The green-shaded region highlights the frequency band of interest (9 to 11 Hz), i.e., the training range of the spatial oscillations. The spectral MSE values corresponding to the phase modulation patterns reported in the first column of Table 1 are 1.109 × 10^−2^ (jointly optimized DL), 1.419 × 10^−1^ (separately optimized DL), 3.576 × 10^−1^ (Fresnel lens array), and 6.243 × 10^−1^ (random diffuser). The jointly optimized diffractive layer consistently achieved the lowest spectral MSE, with more than an order of magnitude improvement over other configurations—all of which used $N_{D}=2.98k$ at the digital backend.

The diffractive layer’s phase profile with a lateral pitch of ~λ/2 and the digital parameters of the backend neural networks were co-optimized through a joint training procedure (detailed in Methods) to maximize the accuracy of the 3D spectral reconstructions. A sequential, three-stage optimization strategy was used: The diffractive layer was first optimized to maximize signal energy and variation (Loss 1); then, the layer and the displacement decoder were jointly optimized (Loss 2); and last, the entire system, including the frequency processor, was trained end-to-end, to maximize spectral reconstruction accuracy (Loss 3). For quantitative comparison, we also used additional optical elements as baseline designs to compare with the jointly optimized diffractive system. Specifically, we evaluated a separately optimized diffractive layer, a Fresnel lens array, and a random phase diffuser (see Methods for details). In this comparison, the separately trained diffractive layer had the same number of optimizable diffractive features, and its surface profile was optimized to efficiently communicate with and focus light onto the output detector array (see Fig. 3A). The backend neural network architecture, including the displacement decoder network and the frequency processor network, remained the same across all the configurations used in this comparison; in these cases, the backend networks were trained separately for accurate reconstruction of the vibration spectra of the structure after the corresponding optical components were fixed.

The performance comparison of these different designs is shown in Fig. 3B, where the input spectra and the reconstructed spectra for each configuration are compared to each other. Performance was quantified using the mean squared error (MSE) of the spectrum within the target frequency range, i.e., 9 to 11 Hz. The performance of the 3D spectral inference for these four configurations is also reported in Table 1 as a function of the number of trainable parameters ($N_{D}$) within the displacement decoder network. The jointly optimized diffractive layer consistently achieved the lowest MSE, with more than an order of magnitude improvement (spectral MSE values are reported in Table 1) over the other configurations across different model capacities. Although some of the alternative configurations showed some predictive capability, they exhibit significantly higher spectral reconstruction errors, as reported in Fig. 3 and Table 1, even with a larger number of trainable parameters in the digital backend. On the other hand, the diffractive vibration monitoring system, with the jointly optimized optical and digital processors, revealed a significantly better 3D spectral inference performance.

**Table 1.** 3D spectral MSE results of different optical configurations, evaluated as a function of the number of trainable parameters (*N*_*D*_) of the displacement decoder network.

Spectral MSE	*N*_*D*_ = 2.98*k*	*N*_*D*_ = 1.75*k*	*N*_*D*_ = 0.85*k*
Diffractive layer (jointly optimized)	1.109 × 10^−2^	1.400 × 10^−2^	1.654 × 10^−2^
Diffractive layer (separately optimized)	1.419 × 10^−1^	1.675 × 10^−1^	2.016 × 10^−1^
Fresnel lens array	3.576 × 10^−1^	3.834 × 10^−1^	4.264 × 10^−1^
Random diffuser	6.243 × 10^−1^	6.545 × 10^−1^	6.760 × 10^−1^

**Fig. 4.**
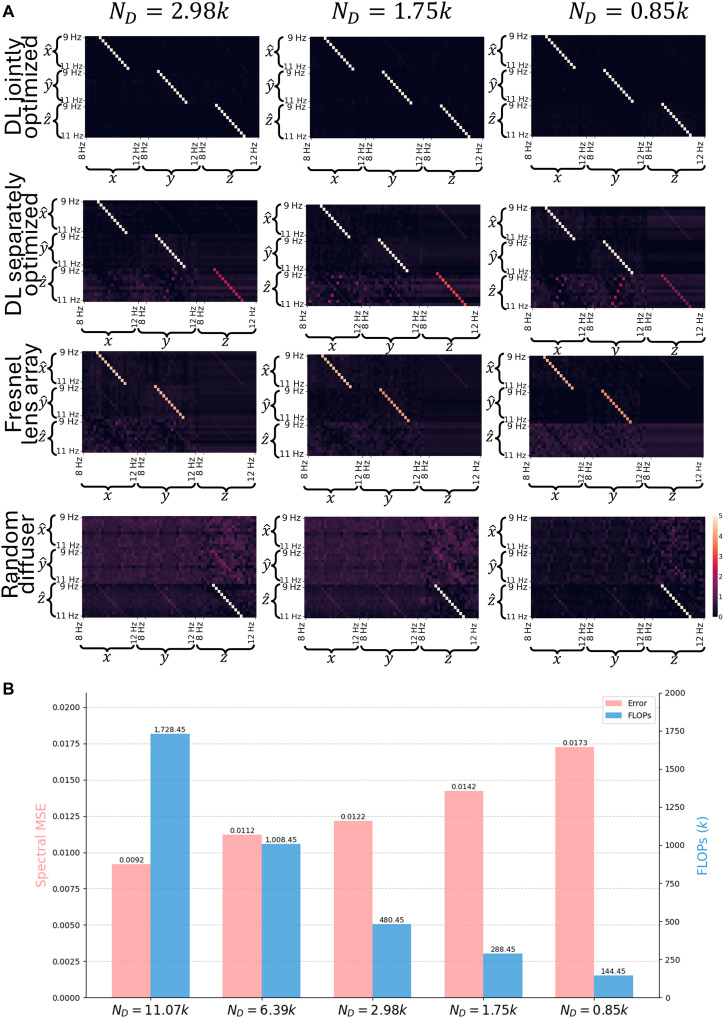
Analysis of 3D oscillation spectra inference performance. (**A**) Confusion matrices for single-frequency inference across various system configurations and displacement decoder sizes ($N_{D}$). The horizontal axis represents input frequencies (ground truth), and the vertical axis represents the inference spectra (9 to 11 Hz) for structural oscillations in *x*, *y*, and *z*. The color bar shows the inference intensity. (**B**) Trade-off between the decoder network complexity and the spectral MSE. Increased model capacity with a larger $N_{D}$ leads to improved spectral accuracy at the cost of an increase in the number of FLOPs needed.

The superior performance of the jointly trained SHM system is further demonstrated through its blind testing performance in single-frequency extraction (in 3D), as detailed in the confusion matrices reported in Fig. 4A. In this analysis, various single-frequency oscillations were applied to the structure under test along one of the directions within the target spectral range. The diffractive system’s inference results, denoted as $\hat{x}$, $\hat{y}$, and $\hat{z}$, corresponding to these single-frequency inputs are shown as columns in the confusion matrix (Fig. 4A). The presence of a clear diagonal in all the configurations of the jointly optimized diffractive layer represents the accurate prediction of these single harmonic oscillations in all three directions (*x*, *y*, and *z*) with minimal cross-talk between neighboring frequencies; on the other hand, significant spectral cross-talk or even complete failures (especially for oscillations in depth, i.e., the *z* direction) are observed in the alternative configurations as shown in the corresponding confusion matrices reported in Fig. 4A. Apart from these 3D analyses, we also obtained similar results for 2D vibration analysis using diffractive processors as reported in figs. S1 and S2 and table S1. Although the joint optimization of the diffractive encoder and the digital backend network demonstrated significant performance advantages, as reported above, a common objective is to develop more compact models that require less computational resources, thereby enhancing potential deployability. This practical consideration motivates a deeper investigation into the relationship between the inference model complexity and performance. To better understand this relationship, we analyzed the trade-off between the displacement decoding network complexity ($N_{D}$) and 3D spectral reconstruction performance. For this analysis, we varied the network’s hidden layer dimensions and examined the correlation between the total floating-point operations (FLOPs) and the resulting 3D spectral MSE. As shown in Fig. 4B, an increased model capacity with a larger $N_{D}$ generally leads to improved spectral inference accuracy, quantifying the performance trade-off between the spectral MSE and the computational cost; these observations are also in agreement with our results reported in Table 1.

To assess the resilience of the diffractive vibration monitoring system against imperfections, we numerically analyzed the impact of surface profile variations on the spectral reconstruction fidelity. These variations were modeled at the pixel level by introducing random height noise to the optimized diffractive layer design, simulating structural discrepancies that may arise from fabrication imperfections/errors or environmental factors. The height noise ($\delta h_{\text{test}}$) at each pixel of the diffractive layer was independently applied following a uniform distribution with a maximum deviation of ±5, ±10, and ±15 μm. To mitigate the potential performance degradation caused by such deviations, we also implemented a “vaccination” strategy during the joint optimization process, where a random height noise ($\delta h_{\text{train}}$) at each pixel of the diffractive layer was dynamically injected into the diffractive layer model during the training phase to condition the system against physical surface irregularities. As illustrated in fig. S3, the “vaccinated” models demonstrated statistically significantly better robustness compared to the standard models that were not vaccinated (with a *P* value of <0.05 under the Welch’s *t* test; reported in table S2). Although the spectral MSE of the standard design increased with the amplitude of $\delta h_{\text{test}},$ the diffractive system that was vaccinated with $\delta h_{\text{train}}∽\left[-10\;\mu m,10\;\mu m\right]$ maintained a high accuracy, achieving a spectral MSE of 1.556 × 10^−2^ even under a noise level of $\delta h_{\text{test}}∽\left[-15\;\mu m,15\;\mu m\right]$. These results indicate that incorporating statistical imperfections into the codesign process might be used to safeguard the output performance against, e.g., manufacturing constraints or potential environmental effects.

Beyond structural surface imperfections or height deviations, we also investigated the sensitivity of the diffractive system to optical alignment errors, specifically variations in the incidence angle of the illumination beam. To quantify this effect, we evaluated the spectral reconstruction performance of a diffractive layer optimized for a nominal incidence angle of $\theta_{\mathrm{inc},\text{train}}=15{}^{\circ}$ across a range of test angles ($\theta_{\mathrm{inc},\text{test}}$). As shown in fig. S4A, the spectral MSE exhibits a minimum at the design angle (i.e., $\theta_{\mathrm{inc},\text{test}}=\theta_{\mathrm{inc},\text{train}}$) and increases as the misalignment grows with $|\theta_{\mathrm{inc},\text{test}}-\theta_{\mathrm{inc},\text{train}}|>0$. However, the system demonstrates a functional degree of angular tolerance; as visualized in fig. S4B, small misalignments (e.g., ±1° shift in illumination angle) result in negligible performance degradation with the reconstructed spectra closely matching the ground truth. Although larger deviations (e.g., more than ±3°) lead to increased spectral distortions, this analysis indicates that the system retains its monitoring capability under minor alignment drifts.

The presented diffractive SHM system comprises four primary components: the illumination source, the diffractive surface, the signal detectors, and the digital backend. The illumination source, operating in either the terahertz or millimeter-wave band, uses an output power of ~40 to 400 mW. For a standard measurement duration of 5 s, this results in a total energy consumption of 200 mJ to 2 J. The diffractive surface functions as a completely passive encoder and consumes no energy during its operation on its own. The voltage-based detector operates as a passive device converting optical signals into voltage; an associated readout circuitry would typically require 7.5 mJ to 0.5 J per measurement cycle. The digital backend, capable of real-time spectrum reconstruction, requires ~72.5 MFLOPs per spectrum reconstruction. Assuming a hardware energy efficiency of 0.5 to 5.5 pJ/FLOP, the digital inference consumes ~0.036 to 0.40 mJ per measurement. Consequently, we can conclude that the total energy consumption of the system is dominated by the illumination source, which can be improved with the use of more efficient sources. Furthermore, the computational latency for spectral reconstruction is ~30 ms on an NVIDIA RTX4090 GPU, which is negligible compared to the signal acquisition time, indicating the system’s capability for real-time monitoring.

### Wavelength multiplexed diffractive systems for multi-point monitoring

**Fig. 5.**
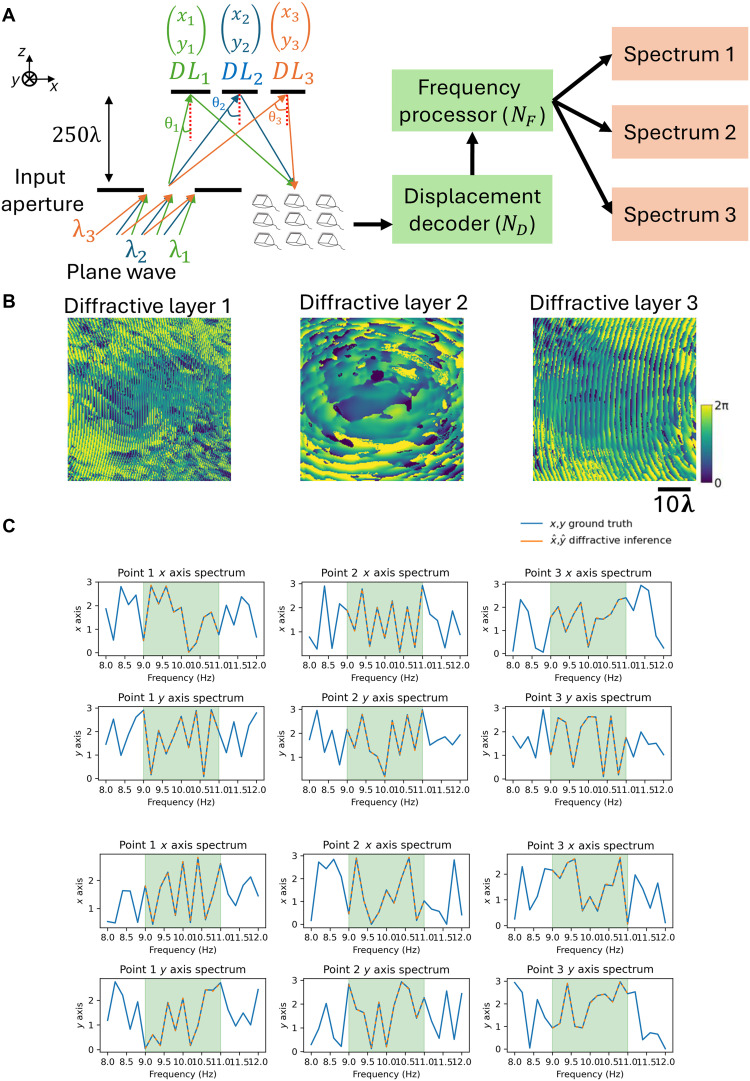
Wavelength-multiplexed diffractive system for simultaneous multipoint vibration monitoring. (**A**) Schematic diagram of the multiplexed sensing configuration. Three coherent input waves with distinct wavelengths (λ_1_, λ_2_, λ_3_) simultaneously illuminate the input aperture at specific incidence angles (θ_1_, θ_2_, θ_3_). These waves propagate over an axial distance to interact with three spatially distinct diffractive layers ($DL_{1},DL_{2},DL_{3}$), each attached to a different desired monitoring point on the structure. The reflected optical fields, modulated by the local 2D displacements ($x_{i},y_{i}$) of each point (including all the cross-talk terms), are collected by a shared detector array. A digital backend, comprising a displacement decoder and a frequency processor, processes the multiplexed signals to simultaneously extract the oscillation spectra at multiple observation points. (**B**) Optimized phase modulation patterns of the three diffractive layers, trained to encode structural vibrations at their respective wavelengths and spatial locations. (**C**) Comparison of the ground truth (solid blue lines) and the diffractive inference results (orange dashed lines) for the 2D oscillation spectra (x and y) across the three monitored points, demonstrating accurate multipoint spectral reconstruction in the target frequency range.

To demonstrate the scalability of the presented framework for high-throughput assessment, we developed a wavelength-multiplexed diffractive system designed to monitor structural vibrations at multiple points simultaneously. As illustrated in Fig. 5A, this multipoint configuration uses three coherent sources with distinct wavelengths (λ_1_ = 0.70 mm, λ_2_ = 0.75 mm, and λ_3_ = 0.80 mm) incident through a common input aperture at incidence angles ($\theta_{\mathrm{inc}}$) of 30°, 45°, and 60°, respectively. The incident waves copropagate to illuminate three independent diffractive layers, each spatially positioned according to the corresponding incidence angle. These spatially distributed diffractive layers modulate the incident wavefronts, including all the wavelengths and cross-talk terms, to encode the local 2D (*x* and *y*) structural displacements at their corresponding locations under test and reflect the optical signals toward a shared detector array, positioned along the reflection path of the illumination waves. The detectors, characterized by a uniform spectral response across the illumination wavelengths, capture the total aggregated energy from the three wavelength channels, generating multiplexed time-series signals. These signals are subsequently processed by a digital backend, where the displacement decoder and frequency processor disentangle the mixed optical information to simultaneously reconstruct the independent vibration spectra for the three monitored points. The phase profiles of the three optimized diffractive layers are depicted in Fig. 5B. The system’s efficacy is further evidenced in Fig. 5C, which presents a comparison between the ground truth and the predicted spectra for the three distinct points that are under test; the close agreement observed in both *x* and *y* oscillation spectra confirms the capability of the jointly optimized system to perform accurate, spectrally multiplexed monitoring of distributed structural locations.

To further characterize the information decoding capacity of this wavelength-multiplexed system, we investigated the relationship between the number of detectors ($N_{\mathrm{dt}}$) and the spectral reconstruction fidelity. To effectively solve this inverse problem, the dimensionality of the measurements must meet or exceed the total degrees of freedom being monitored—specifically, the product of the number of monitored spatial points and the number of vibration axes per point. In our setup, simultaneously monitoring 2D vibrations (*x* and *y*) across three distinct locations establishes 6 degrees of freedom. Therefore, we evaluated the system’s performance using detector arrays with $N_{\mathrm{dt}}=4,6$, and 9 (illustrated in table S3) while maintaining identical training strategies and digital backend architectures (model capacity) to ensure a controlled and fair comparison. As shown in fig. S5A, the configuration with $N_{\mathrm{dt}}=4$ represents an underdetermined system; consequently, it failed to accurately disentangle the multiplexed spectral features, resulting in a relatively high spectral MSE of 1.446 × 10^−1^ ± 4.646 × 10^−2^ (means ± SD). In contrast, when the number of detectors was increased to meet or exceed the required degrees of freedom, i.e., $N_{\mathrm{dt}}\geq 6$, the system successfully recovered the vibration spectra. As demonstrated in fig. S5 (B and C), the architectures with $N_{\mathrm{dt}}=6$ and $N_{\mathrm{dt}}=9$ achieved accurate spectral inference with significantly reduced MSE values of 5.276 × 10^−4^ ± 7.224 × 10^−4^ and 3.555 × 10^−4^ ± 2.772 × 10^−4^, respectively. The statistical significance of this performance improvement as a function of $N_{\mathrm{dt}}$ is also reported in table S3. These results confirm that accurate, simultaneous multipoint monitoring is achievable provided that the detector count is scaled to accommodate the dimensionality of the target structural dynamics.

To better quantify the advantages of spectral diversity in high-throughput multipoint monitoring, we also compared the performance of the wavelength-multiplexed system against a baseline monochrome configuration (shown in fig. S6A) where all three diffractive layers were illuminated by the same wavelength, also through a common input aperture at incidence angles ($\theta_{\mathrm{inc}}$) of 30°, 45°, and 60°—same as before. Stated differently, in this baseline comparison, illumination angle diversity for multipoint monitoring was kept while the wavelength multiplexing was dropped out to better quantify its impact on the output performance. As illustrated in fig. S6, despite using an identical detector array geometry ($N_{\mathrm{dt}}=9$), the monochrome system (fig. S6B) exhibited increased deviations (spectral MSE 7.175 × 10^−4^ ± 4.368 × 10^−4^) from the ground truth multipoint spectra. Conversely, the wavelength-multiplexed system (fig. S6C) achieved a statistically significantly better reconstruction fidelity (spectral MSE 3.555 × 10^−4^ ± 2.772 × 10^−4^, with a *P* value of 2.196 × 10^−10^) compared to the monochrome setup. This quantitative comparison highlights that spectral diversity serves as a powerful encoding dimension, allowing the digital backend to effectively disentangle mixed vibration signals from shared detectors and enabling accurate parallel monitoring of multiple target points on a structure, significantly outperforming monochrome spatial multiplexing.

### Experimental demonstration of diffractive vibration monitoring

**Fig. 6.**
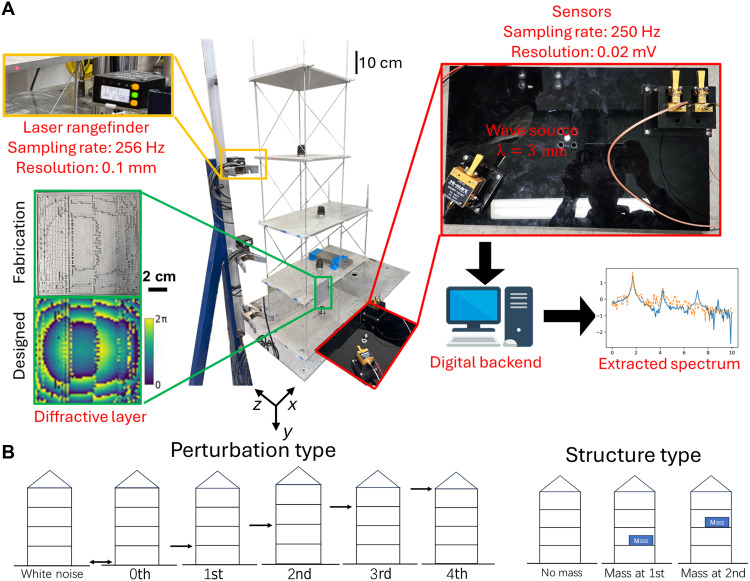
Experimental validation of the 1D diffractive vibration monitoring system using a millimeter-wave (λ = 3 mm) source. (**A**) Photograph of the experimental setup and the pipeline for 1D spectrum inference. (**B**) Schematic of the types of perturbations and structures tested in these experiments.

We experimentally validated the presented concept using 3D-printed diffractive layers and millimeter-wave illumination. An initial experimental setup, depicted in Fig. 6A, was designed to monitor the 1D (*x* axis) oscillations of a test structure, which was a four-level building model with its base floor mounted on a programmable shake table for controlled perturbations. This experimental validation is not only a performance check but also a demonstration of how physical encoding combined with minimal-data processing can operate under noisy, nonideal conditions—a key benchmark for deployment in uncontrolled environments. To suppress displacements in the *z* direction and concentrate motion primarily along the *x* axis, metal wires were used to constrain the model along the *z* axis, effectively increasing its stiffness in that direction. Ground truth displacement data for the test structure were simultaneously acquired using laser rangefinders. The core of the diffractive vibration monitoring system involved an optimized, 3D-printed diffractive layer, which was affixed to the first level of the building model (Fig. 6A). A coherent source at λ = 3 mm illuminated the structure, and the reflected waves were spatiotemporally modulated by the displacements of the passive diffractive layer on the structure; these reflected signals were captured by two (single-pixel) detectors (see the Methods section). During these measurements, various excitation methods were used to induce different structural vibrations (shown in Fig. 6B), including applying a white noise signal to the shake table, programmed shaking profiles of the base floor, and manual perturbations to the base, first, and upper levels. Furthermore, to simulate variations in the building structure, a mass block was systematically placed and fixed at different levels, altering both the mass distribution and the dynamic response of the system, thereby changing its natural frequency.

**Fig. 7.**
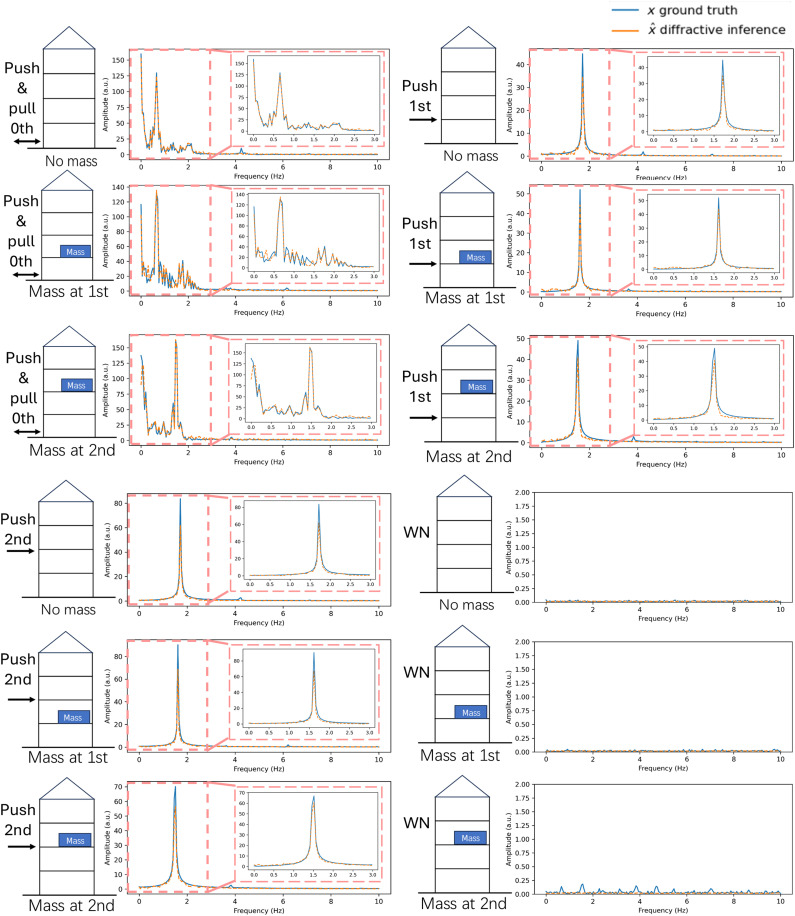
Experimental results of the 1D diffractive vibration monitoring system using a millimeter-wave source. Spectral inference results of different configurations with various types of perturbations and structures are compared against the ground truth. WN, white noise; a.u., arbitrary units. The nonzero energy at zero frequency was caused by the displacement of the base level (zeroth level) due to the manual perturbation. Also see figs. S7 and S8 for additional experimental results.

The temporal signals captured by the two detectors were decoded by a trained digital backend, which predicted the frequency spectra of the structure’s vibrations within a predefined spectral range of interest. Figure 7 and fig. S7 compare the ground truth spectra (measured by a laser rangefinder) and the spectra extracted by the diffractive system for various structures with different mass placements and perturbations. The accurate predictions of both the vibration frequencies and their corresponding amplitudes in these experimental results confirm the feasibility and effectiveness of the diffractive system for monitoring structural dynamics. We also compared the inference performance of the optimized diffractive layer to that of a reflective flat mirror, as depicted in fig. S8. In this comparison, both sets of the digital backend neural networks were trained/optimized using an identical number of measurements with the same network architecture (i.e., with the same number of trainable parameters) to provide a fair comparison. The configuration with the optimized diffractive layer yielded a significant improvement in its spectral MSE results over the results achieved with a flat mirror, which stems from the optimized diffractive layer’s ability to interact more effectively with the output detectors. Compared to a flat mirror or another reflective optical element, an optimized diffractive layer enhances the SD of the detected signals under various structural vibrations. This results in an enhanced 3D oscillation encoding capability through the diffractive layer because a higher SD in the detected signals means that different oscillation spectra produce more distinguishable intensity distributions across the output detectors, making it easier for the jointly optimized digital decoder to learn the mapping required for accurate spectral reconstructions.

**Fig. 8.**
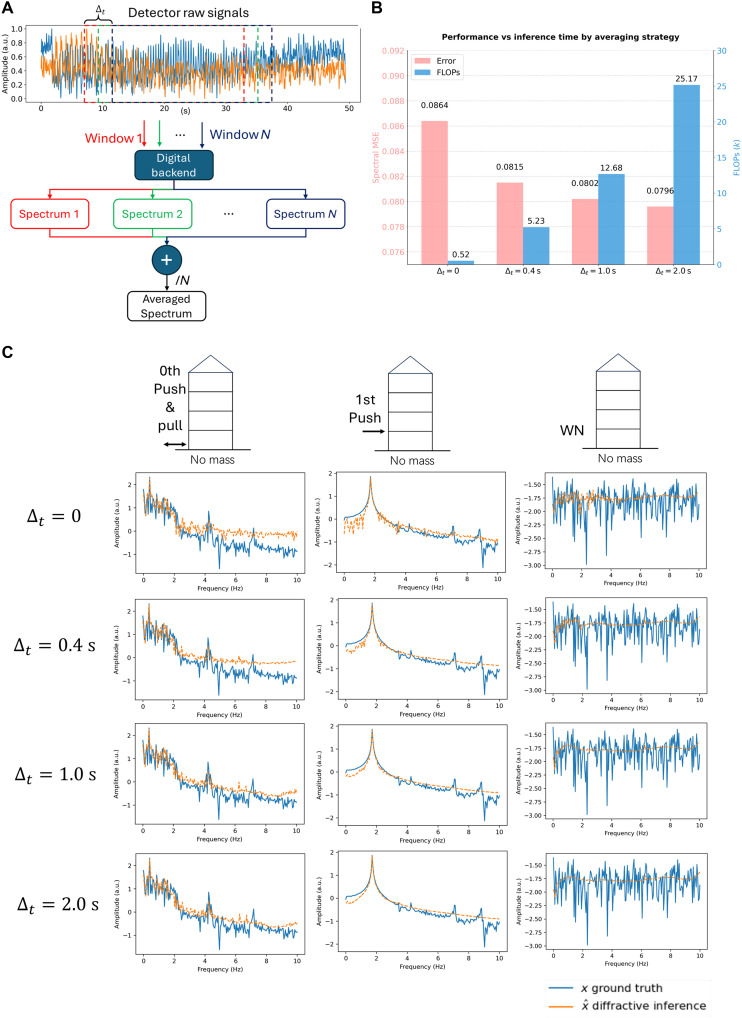
Temporal averaging improves spectral inference fidelity in diffractive vibration monitoring. (**A**) Schematic of the temporal averaging method: Raw sensor signals are processed using a sliding window to generate multiple time-series segments. Each segment’s spectrum, extracted by the nonlinear digital backend, is then averaged to produce the final spectral output. (**B**) Trade-off between the spectral inference accuracy and the computational cost. (**C**) Comparison of the extracted spectra (amplitude versus frequency) for different temporal averaging times ($\Delta_{t}$) under three different experimental conditions. WN, white noise. The nonzero energy at zero frequency was caused by the displacement of the base level (zeroth level) due to manual perturbation.

To further improve the spectral inference accuracy of our approach, we developed a postprocessing method based on temporal averaging, outlined in Fig. 8A. This strategy uses a sliding window of constant duration across each detector’s temporal signal stream to generate multiple (potentially overlapping) time-series segments. The range of the sliding windows is defined by the temporal averaging time $\Delta_{t}$, representing the total temporal span across which the centers of the sliding windows are distributed for the averaging calculation. Each segment serves as an individual input to the trained digital backend, which extracts the oscillation spectrum associated with that time window. The final spectral output is then computed by averaging the ensemble of vibration spectra extracted from all of the time segments. The inherent trade-off between spectral MSE and the cumulative FLOPs is depicted in Fig. 8B; averaging over a greater number of temporal windows improved the spectral inference accuracy (lowering the spectral MSE) while also increasing the required computational time. Figure 8C provides a comparison of the extracted spectra for different $\Delta_{t}$ values, illustrating the impact of this approach on the quality of our spectral reconstructions.

**Fig. 9.**
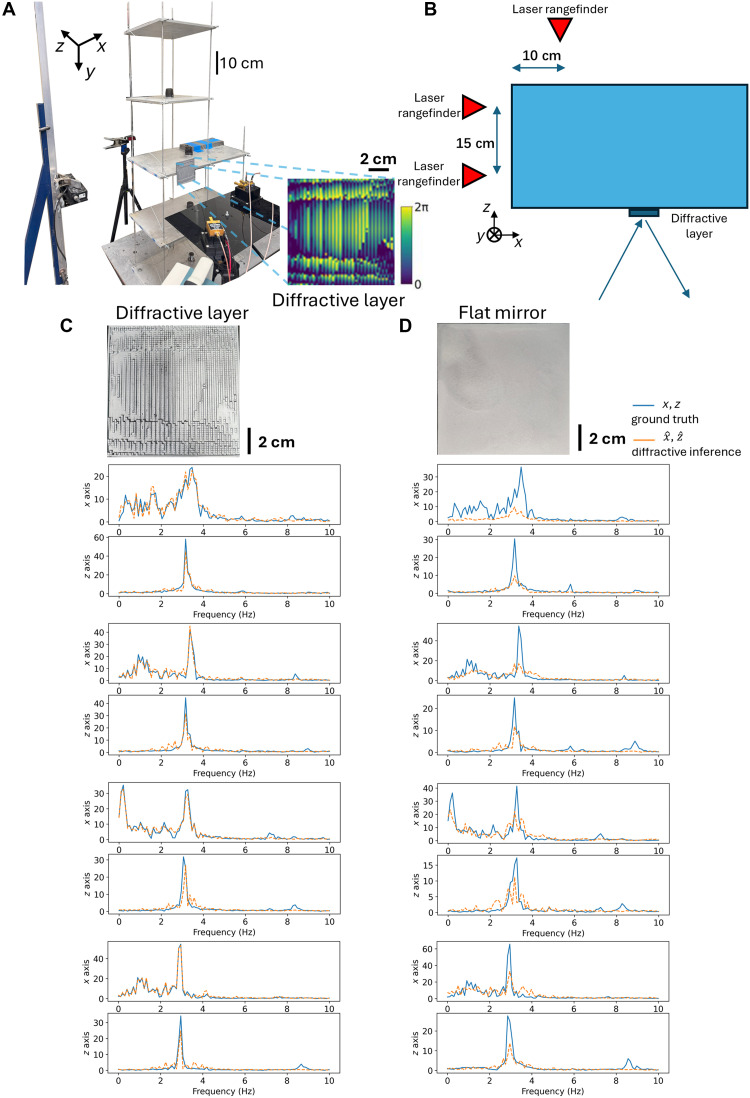
Experimental validation of the 2D vibration monitoring system using a millimeter-wave source. (**A**) Photograph of the experimental setup for 2D vibration spectrum inference. (**B**) Top-down schematic view showing the placements of the laser rangefinders (used for ground truth measurements) and the diffractive layer. Experimental performance comparison of the 2D diffractive vibration monitoring system between (**C**) the optimized diffractive layer and (**D**) a reflective flat mirror that are both 3D printed. The nonzero energy at zero frequency was caused by the displacement of the base level (zeroth level) due to the manual perturbation.

We further investigated the diffractive processor-based vibration monitoring system by measuring 2D (*x* and *z*) oscillations of the same building model (see Fig. 9A). For these experiments, the restraining wires that were previously used to prevent motion along the *z* axis were loosened to allow for 2D displacements of the test structure. Laser rangefinders were installed on adjacent sides of the first level (shown in Fig. 9B) to provide reference (ground truth) measurements of 2D displacements. Another optimized diffractive layer (shown in Fig. 9A), mounted on the same level, was used to spatiotemporally modulate the incident wave in accordance with the structure’s 2D vibrations. The test structure was subjected to 2D oscillations using a programmable shake table that reproduced seismic waveforms in the *x* direction from the NGA-West2 earthquake dataset (*48*), applied at both full scale and 0.1x scale while the perturbations of the *z* axis were introduced by pushing manually (details in Methods). To rigorously evaluate the system’s generalization capabilities, we collected data from 20 independent earthquake records and partitioned the dataset into nonoverlapping training (80%) and testing (20%) subsets. Consequently, the results presented here are derived exclusively from the testing subset, representing out-of-distribution (OOD) performance on unique seismic signals corresponding to new earthquakes that were not seen during the training of the digital backend. The diffractive processor-based system simultaneously extracted the spectra of the building’s displacements in both *x* and *z* directions from the two detector signals. Examples of spectral inference results for *x* and *z* directions are visualized in Fig. 9C, along with the corresponding ground truth measurements. The performance advantages of the optimized diffractive layer compared to the 2D inference results obtained with a flat mirror are also illustrated in Fig. 9D, supporting the same conclusions as in our earlier experiments reported in fig. S8.

The performance advantages observed through the joint optimization of the diffractive layer and the digital backend extends beyond mere accuracy improvements; it represents a new approach to how sensing systems can be designed. By codesigning the passive optical encoder material with the subsequent shallow neural network decoder, the system implicitly learns to transform complex, multidimensional structural oscillation information into highly distilled, yet robustly decodable, optical signals that can be captured by a minimal number of detectors. This optical preprocessing effectively offloads notable computational burden from the digital domain to the physical layer, enabling the use of shallow and low-power neural networks for rapid and accurate spectral reconstruction, a capability unattainable with traditional sensing architectures that would require far more detectors and extensive postprocessing for comparable performance. This fundamental principle of learned physical-digital compression holds transformative potential for numerous sensing applications where data efficiency, low power, and remote operation are critical constraints, such as in autonomous navigation and distributed environmental sensing. The synergy between the optimized physical-layer encoding and the efficient neural network decoding signifies a move toward “intelligent sensing at the source,” where essential information is extracted and compacted through light-matter interactions before traditional digital processing takes over. This minimizes data and computational overhead, addressing a critical bottleneck in ubiquitous, high-density sensor deployments and laying the groundwork for truly distributed, low-latency monitoring networks.

## DISCUSSION

We developed a hybrid system, integrating a jointly optimized diffractive layer with a shallow digital neural network backend for rapidly extracting structural vibration frequency spectra. The effectiveness of this hybrid system was demonstrated through both numerical simulations and experiments using millimeter-wave illumination. We observed major performance improvements through the joint optimization of the diffractive layer and the backend neural networks; this jointly optimized system not only significantly outperformed other optical configurations with nontrainable optical elements such as flat mirrors, Fresnel lens arrays, or random diffusers but also showed major performance improvements compared to separately optimized diffractive layers. These observations can be attributed to the jointly optimized diffractive layer’s ability to effectively encode 3D structural vibrations into the optical wavefront, enhancing signal variations at the output detectors and creating more distinguishable spatiotemporal patterns in response to various structural perturbations, helping the decoder backend to reveal the 3D oscillation spectra accurately.

The use of trainable diffractive optics as a front-end signal encoder, paired with a jointly trained neural decoder, introduces a new strategy for compact, low-data-rate sensors that can operate without dense sampling or extensive storage. SHM methods are commonly categorized on the basis of their measurement approach, including vibration-based, static or quasistatic, acoustic or ultrasonic, and optical or imaging-based techniques. The proposed framework falls under the optical sensing category but introduces a fundamentally different mechanism for vibration acquisition and interpretation. Traditional optical systems, such as laser vibrometry, offer high accuracy but are limited by alignment requirements, line-of-sight access, and power consumption. Vibration-based and static networks provide reliable local data but require dense deployment and wiring, increasing installation and maintenance costs. Vision-based systems can capture distributed motion fields but are sensitive to lighting and computationally intensive. In contrast, the presented diffractive system uses a spatially optimized passive layer that encodes 3D vibrations into modulated light, rapidly and efficiently decoded in real time by a shallow and low-power neural network. Although substantially improving scalability, energy efficiency, robustness, and multiplexing capabilities, it maintains comparable accuracy and frequency resolution. Consequently, it unifies the strengths of high-performance optical sensing and scalable vibration-based monitoring, advancing SHM toward a more energy-efficient and data-efficient paradigm. The significance of this work extends beyond potential performance gains in SHM. Its hybrid design paradigm may inspire previously unrealized classes of passive sensors in disciplines such as aerospace engineering and robotics, where power and size constraints preclude conventional sensing architectures.

Although diffractive neural networks have shown promise in computational imaging and classification of static objects, their application to sensing dynamic physical systems remains nascent. This work moves beyond static inference tasks by demonstrating a system that leverages a passive diffractive encoder, co-optimized with a temporal decoder, to extract complex, time-varying 3D spectral information from a moving object. Unlike standard end-to-end optimization approaches, this is achieved through a hierarchical training scheme that first maximizes physical signal contrast before jointly optimizing for temporal displacement decoding. Therefore, another conceptual advancement of our work is this joint physical-digital optimization strategy that simultaneously encodes multi-point 3D structural dynamics—a problem space with unique challenges, such as continuous-time signals from multiaxis and multipoint displacements—into a low-data-rate optical stream.

Another critical advantage of the presented framework is its inherent generalizability, which eliminates the need for structure-specific retraining or hardware refabrication. This scalability arises from the system’s hierarchical architecture, which decouples motion sensing from structural diagnostics; the diffractive layer and the decoder are jointly optimized to map optical signals directly into quantitative 3D displacement time series—a physical mapping independent of structural identity. By using a training curriculum based on randomized harmonic oscillations rather than specific building dynamics, the system functions as a general-purpose motion sensor capable of extracting vibration spectra from diverse structures without modifications of its architecture.

Beyond demonstrating the core functionality of diffractive vibration monitoring systems, this work also investigated practical trade-offs influencing the system’s performance and computational demands. We quantitatively explored the relationship between the complexity of the digital backend network and spectral reconstruction accuracy by varying the hidden layer dimensions of the displacement decoding network. As illustrated in Fig. 4B, using decoder networks with more trainable parameters, corresponding to higher numbers of FLOPs, generally leads to reduced 3D spectral MSE. In addition, we introduced and experimentally validated a temporal averaging-based postprocessing strategy, which leverages multiple time segments from the sensor stream to enhance the final spectral reconstruction fidelity significantly. These findings provide valuable insights for tailoring the diffractive system’s configuration to specific application requirements, balancing accuracy, latency, and available computational power. A key aspect of the temporal averaging-based postprocessing method is its departure from traditional linear operations, such as those used in standard periodograms. Our technique uses a nonlinear neural network-based displacement decoder, which interacts differently with the diverse features present in individual segments of the time-sequence detector data. This processing through the network’s architecture underpins a complex N-to-N nonlinear mapping from the raw temporal segments to their respective spectral estimates. Such temporally shifted successive nonlinear mappings offer distinct advantages, particularly in the capacity to more effectively mitigate noise and handle unexpected or OOD data points that may be present in the measured time signals.

Although our experimental validation at millimeter-wave frequencies on a laboratory-scale model demonstrates the foundational principles and remarkable efficacy of this diffractive vibration monitoring system, the path toward widespread real-world deployment also presents unique challenges and opportunities that warrant careful consideration. Translating the millimeter-wave results to optical or infrared (IR) wavelengths for civil infrastructure applications will necessitate the development of large-area, robust diffractive layers with feature sizes on the order of hundreds of nanometers, resilient to environmental factors such as temperature fluctuations, humidity, and structural deformation over long periods. Furthermore, ensuring the long-term stability and calibration of both the diffractive layer and the remote illumination/detection system in dynamic outdoor environments will be crucial for maintaining the high accuracy demonstrated in controlled settings. The development of self-calibrating mechanisms or adaptive learning algorithms to compensate for environmental drift and material degradation will be important steps toward robust field implementations.

A major future direction in diffractive SHM systems involves scaling this technology for higher throughput assessment of structures by monitoring numerous locations simultaneously. Extending the current approach for this goal could involve spatial multiplexing, where multiple codesigned monochrome diffractive vibration processors monitor different points, potentially feeding data into a unified backend neural network. An even more advanced concept involves spectral-spatial multiplexing, assigning distinct wavelengths to different monitored locations of a structure, each potentially using a specifically tuned diffractive layer and detector array, allowing for potentially higher density of structures to be monitored in parallel and processed by a common backend decoder.

The exploration of shorter operational wavelengths, particularly within the visible and IR spectra, presents a compelling avenue for enhancing the accuracy and resolution of diffractive SHM systems due to the scalability of diffractive optical processors with respect to the wavelength of illumination. Although the presented work experimentally validated the diffractive SHM systems using millimeter waves, the underlying principles of diffractive layer design allow for scalability across different parts of the electromagnetic spectrum by adjusting the dimensions of the diffractive features proportional to the illumination wavelength. Transitioning the illumination wavelengths to the visible or IR spectrum would require the fabrication of diffractive elements with substantially smaller lateral feature sizes. This refinement in feature resolution, achievable with advanced 3D manufacturing techniques such as two-photon polymerization and optical lithography that provide submicrometer fabrication precision, offers the potential for more precise wavefront modulation (*49*–*51*). Furthermore, to ensure robustness against inevitable fabrication tolerances at these reduced feature sizes, a “vaccination” strategy (detailed in the Results section) can be used. By explicitly incorporating various forms of noise, distortions or imperfections into the training schedule, the diffractive layers can be optimized to remain resilient to manufacturing imperfections. The capability to produce such fine structures is critical for operating effectively at shorter wavelengths and could lead to substantial improvements in the sensitivity and overall accuracy of diffractive SHM systems. These advances could pave the way for a new generation of high-accuracy, compact, multiplexed and more cost-effective SHM solutions.

Given its foundation in diffractive optics and neural computation, our system is inherently adaptable to other spectral bands, functional targets, and time-varying environments. The potential to extend this approach into the visible or near-infrared regime—enabled by nanoscale fabrication—could enable ultracompact sensors for environmental monitoring or industrial robotics. We envision that the physical encoding strategies described here will find broader applications across science and engineering, enabling systems where sensing and computation are not considered separately but co-optimized within the same physical substrate.

## METHODS

### Numerical forward model

In the design of a diffractive vibration monitoring system, the optical forward model can be depicted by two successive operations: (i) shifted free-space propagation of the optical field and (ii) wave modulation by the reflective diffractive layer. The shifted free space propagation for an axial distance *d* and lateral shifting distance $x_{0},y_{0}$ of the complex field u(x,y) was calculated using the shifted angular spectrum approach (*52*) and can be written as

$${\mathbb{P}}_{x_{0},y_{0},d}u\left(x,y\right)=F^{-1}\left\{F\left\{u\left(x,y\right)\right\}\;{\hat{H}}^{\prime }\left(f_{x},f_{y};x_{0},y_{0},d\right)\right\}$$ (1)

where ${\mathbb{P}}_{x_{0},y_{0},d}$ represents the free-space propagation operator for a lateral shift of $\left(x_{0},y_{0}\right)$ and an axial distance of *d*, F, and $F^{-1}$ are the 2D Fourier transform and the inverse Fourier transform operators, respectively. ${\hat{H}}^{\prime }\left(f_{x},f_{y};x_{0},y_{0},d\right)$ is the transfer function of free space, defined as

$${\hat{H}}^{\prime }\left(f_{x},f_{y};x_{0},y_{0},d\right)=\hat{H}\left(f_{x},f_{y};x_{0},y_{0},d\right)\text{rect}\left(\frac{f_{x}-f_{x,0}}{f_{x,\text{width}}}\right)\text{rect}\left(\frac{f_{y}-f_{y,0}}{f_{y,\text{width}}}\right)$$ (2)

$$\begin{array}{c} \hat{H}\left(f_{x},f_{y};x_{0},y_{0},d\right)=\\ \left\{ \begin{array}{cc} \text{exp}\left\{j2\pi \left(d\sqrt{\lambda^{-2}-f_{x}^{2}-f_{y}^{2}}+x_{0}\;f_{x}+y_{0}\;f_{y}\right)\right\}, & f_{x}^{2}+f_{y}^{2}<\frac{1}{\lambda^{2}}\\ 0, & f_{x}^{2}+f_{y}^{2}\geq \frac{1}{\lambda^{2}} \end{array}\right. \end{array}$$ (3)

where $k=\frac{2\pi }{\lambda }$ and λ is the wavelength of the light. $f_{x}$ and $f_{y}$ are the spatial frequencies along the x and y directions, respectively. rect(·) is the unit rectangular function that is used as a bandpass filter to avoid aliasing errors (*52*). The central frequency $f_{\left\{x,y\right\},0}$ and bandpass width $f_{\left\{x,y\right\},\text{width}}$ can be calculated by

$$f_{\left\{x,y\right\},0}=\left\{ \begin{array}{cc} \frac{f_{\left\{x,y\right\},\text{limit}}^{\left(+\right)}+f_{\left\{x,y\right\},\text{limit}}^{\left(-\right)}}{2}, & {\left(2\Delta f_{\left\{x,y\right\}}\right)}^{-1}\leq {\left\{x,y\right\}}_{0}\\ \frac{f_{\left\{x,y\right\},\text{limit}}^{\left(+\right)}-f_{\left\{x,y\right\},\text{limit}}^{\left(-\right)}}{2}, & -{\left(2\Delta f_{\left\{x,y\right\}}\right)}^{-1}<{\left\{x,y\right\}}_{0}<{\left(2\Delta f_{\left\{x,y\right\}}\right)}^{-1}\\ -\frac{f_{\left\{x,y\right\},\text{limit}}^{\left(+\right)}+f_{\left\{x,y\right\},\text{limit}}^{\left(-\right)}}{2}, & {\left\{x,y\right\}}_{0}\leq -{\left(2\Delta f_{\left\{x,y\right\}}\right)}^{-1} \end{array}\right.$$ (4)

$$f_{\left\{x,y\right\},\text{width}}=\left\{ \begin{array}{cc} f_{\left\{x,y\right\},\text{limit}}^{\left(+\right)}-f_{\left\{x,y\right\},\text{limit}}^{\left(-\right)}, & {\left(2\Delta f_{\left\{x,y\right\}}\right)}^{-1}\leq {\left\{x,y\right\}}_{0}\\ f_{\left\{x,y\right\},\text{limit}}^{\left(+\right)}+f_{\left\{x,y\right\},\text{limit}}^{\left(-\right)}, & -{\left(2\Delta f_{\left\{x,y\right\}}\right)}^{-1}<{\left\{x,y\right\}}_{0}<{\left(2\Delta f_{\left\{x,y\right\}}\right)}^{-1}\\ f_{\left\{x,y\right\},\text{limit}}^{\left(-\right)}-f_{\left\{x,y\right\},\text{limit}}^{\left(+\right)}, & {\left\{x,y\right\}}_{0}\leq -{\left(2\Delta f_{\left\{x,y\right\}}\right)}^{-1} \end{array}\right.$$ (5)

where $\Delta f_{\left\{x,y\right\}}$ is the sampling frequency in the {x,y} axis, and the limit spatial frequency values are calculated by

$$f_{\left\{x,y\right\},\text{limit}}^{\left(\pm \right)}={\left[{\left({\left\{x,y\right\}}_{0}\pm \frac{1}{2\Delta f_{\left\{x,y\right\}}}\right)}^{-2}d^{2}+1\right]}^{-\frac{1}{2}}\lambda^{-1}$$ (6)

The optimizable diffractive layer (*53*–*57*) is modeled as a reflective diffractive optical element that modulates the phase of the incident wavefront. The reflectance coefficient r(x,y) of the diffractive layer can be written as

$$r\left(x,y\right)=\text{exp}\left[j\phi \left(x,y\right)\right]$$ (7)

where ϕ(x,y) is the phase modulation function of the trainable diffractive layer. The reflectance coefficient of the shifted diffractive layer can be calculated as

$$\begin{array}{c} r_{\text{shifted}}\left(x,y,x_{d,0},y_{d,0}\right)=\text{exp}\left[j\phi \left(x-x_{d,0},y-y_{d,0}\right)\right]=\\ F^{-1}\left(F\left\{\text{exp}\left[j\phi \left(x,y\right)\right]\right\}\times \text{exp}\left[-j2\pi \left(f_{x}x_{d,0}+f_{y}y_{d,0}\right)\right]\right) \end{array}$$ (8)

where $x_{d,0},y_{d,0}$ represent the lateral displacement of the diffractive layer.

The forward model for multipoint vibration monitoring (see Fig. 5 and figs. S5 and S6) was extended to simulate the simultaneous illumination of the spatially distributed diffractive layers by multiple incident wavefronts. In both the wavelength-multiplexed and monochrome illumination-based multipoint monitoring, the model explicitly accounted for optical cross-talk by calculating the interaction of each incident wave with the complete array of diffractive layers, rather than treating each layer in isolation. For the wavelength-multiplexed configuration, coherence was maintained within each wavelength channel; the complex optical fields reflected from all the diffractive layers under a single illumination wavelength ($\lambda_{i}$) were first coherently added to determine the field distribution for that channel; the total intensity at the detector plane was then computed as the linear sum of the intensities resulting from the distinct wavelength channels. For the single-wavelength (monochrome) configuration, where all the incident waves share the same wavelength, the system was modeled as a fully coherent architecture. In this case, the complex fields resulting from all the illumination angles and diffractive layer interactions were summed before the final intensity calculation, thereby incorporating the interference effects arising among all the reflected and propagating wavefronts.

The Fresnel lens array was generated by stitching four Fresnel lens phase patterns, each aligned such that the center of the corresponding sensor coincided with the focus point. The Fresnel lens phase pattern was calculated by

$$\phi_{\text{Fresnel}}=-\frac{2\pi }{\lambda }\left(\sqrt{f^{2}+x^{2}+y^{2}}-f\right)$$ (9)

where f is the focal length of the Fresnel lens.

### Network structure for the digital backend

The digital backend network used in spectral reconstructions is composed of a displacement decoder network and a frequency processor network. The displacement decoder network extracts the measured signals into the structural displacements in different directions, whereas the frequency processor network extracts the oscillation spectra in the target range from the extracted displacement in different directions. The displacement decoder network was composed of three fully connected layers with ReLU (rectified linear unit) activation. The parameter $N_{D}$ denotes the number of trainable parameters of the displacement decoder network, e.g., $N_{D}=2.98k$ for the network used for 3D spectral reconstructions with the input dimension $N_{\mathrm{dt}}=4$, the hidden layer dimension $N_{h}=\left[64,32,16\right]$ and the output dimension $N_{o}=3$. For the frequency processor network, $N_{F}$ denotes the number of trainable parameters, and an initial Fast Fourier Transform (FFT) layer converts the time-domain signal to the frequency domain, after which a single-layer perceptron (without activation) extracts the desired spectral band in different directions (*x*, *y*, and *z*).

For the simultaneous multipoint vibration monitoring tasks, the architecture of the displacement decoder network was expanded to accommodate the increased dimensionality of the inverse problem. This multipoint network comprised five fully connected hidden layers with dimensions of $N_{h}=\left[256,128,64,32,16\right]$ with $N_{D}=46.42k$. To investigate the relationship between the measurement sparsity and the decoding performance, the input dimension of the network was varied on the basis of the number of detectors used in the array, specifically evaluating configurations with $N_{\mathrm{dt}}\in \left\{4,6,9\right\}$. The final output layer dimension was set to $N_{o}=6$, allowing for the simultaneous prediction of the *x* and *y* displacements as a function of time for all three monitored locations on the structure of interest.

### Training data preparation

The displacements along the axes were synthesized by a linear combination of harmonic oscillations in the target frequency window. The amplitudes of the harmonic oscillations were randomly generated with an amplitude upper bound of $A_{\max}$

$$A_{\left\{x,y,z\right\},j}∼U\left[0,A_{max}\right]$$ (10)

where $A_{\max}$ was set as ∼1.5λ whereas the phase values of the harmonic oscillations $\phi_{\left\{x,y,z\right\},j}$ were randomly generated between 0 and 2π. The displacement of the diffractive layer at time stamp *t* was calculated using

$$\Delta {\left\{x,y,z\right\}}_{t}=\sum_{j=1}^{M}A_{\left\{x,y,z\right\},j}\text{sin}\left(2\pi f_{j}t+\phi_{\left\{x,y,z\right\},j}\right)$$ (11)

where M represents the total number of discrete frequencies considered. $f_{j}$ denotes the *j*th frequency component, selected from the discrete set {8.0, 8.2, …, 11.8, 12.0} Hz.

### Training scheme and loss function

The whole model, including the diffractive layer, displacement decoder network, and frequency processor network, was trained for 200 epochs. In the first 10 epochs, only the diffractive layer was optimized with the following loss function

$${\text{loss}}_{1}=\alpha_{1}\left(1-\frac{1}{K}\sum_{t=1}^{K}\sum_{i=1}^{D}s_{i,t}\right)+\alpha_{2}\left[1-std\left(1_{D}^{T}\cdot S\right)\right]$$ (12)

where K is the number of time sampling points, D is the number of detectors, and $S\in R^{D\times K}$ where the element in the *i*th row and *t*th column is denoted as $s_{i,t},$ representing the ith detector reading value at the sampling time t, normalized by the total energy of the input wave. $1_{D}^{T}$ is a 1×D matrix (a row vector) with all entries equal to 1. std(·) is the SD function, which is used to maximize the intensity variations on the detectors when the structure under test is oscillating in the target range of frequencies. $\alpha_{1}$ and $\alpha_{2}$ are hyperparameters, which are both empirically set as 1.

Between the 11th epoch and the 100th epoch, the diffractive layer and displacement decoder network were jointly optimized using the following loss function

$${\text{loss}}_{2}={\text{loss}}_{1}+\alpha_{3}\mathrm{MSE}\left[\text{DispNet}\left(S\right),\{\Delta x_{t},\Delta y_{t},\Delta z_{t}\}\right]$$ (13)

where DispNet(·) is the displacement decoder network predicting the displacement at different directions (*x*, *y*, and *z*), MSE(·) is the MSE function, and $\Delta x_{t},\Delta y_{t}$, and $\Delta z_{t}$ are the ground truth displacement vectors for *x*, *y*, and *z*, respectively. $\alpha_{3}$ is a hyperparameter empirically set as 0.1.

During the 101st epoch to the 200th epoch, the diffractive layer, displacement decoder network and the frequency processor network were jointly trained using the following loss function

$${\text{loss}}_{3}={\text{loss}}_{2}+\alpha_{4}\mathrm{MSE}\left\{\text{FreqNet}\left[\text{DispNet}\left(S\right)\right],\{A_{x},A_{y},A_{z}\}\right\}$$ (14)

where FreqNet(·) is the frequency processor network that transforms the extracted temporal displacement into the frequency domain within the predetermined spectral range of interest, and $A_{x},A_{y}$, and $A_{z}$ are the ground truth oscillation amplitudes in the frequency domain along the *x*, *y*, and *z* directions, respectively. $\alpha_{4}$ is a hyperparameter that is empirically set as 1.

In our comparative analyses, the separately optimized diffractive layer was first trained over 10 epochs with the same strategy as the training of the jointly trained diffractive system, using the same loss function as described in Eq. 12. Starting from the 11th epoch, the digital backends for the separately optimized diffractive layers were trained for 190 epochs (i.e., for the same number of epochs used in the training of the digital backends of the jointly trained configuration). Until the 100th epoch, the displacement decoder network was trained using the following loss function

$${\text{loss}}_{4}=\alpha_{3}\mathrm{MSE}\left[\text{DispNet}\left(S\right),\{\Delta x_{t},\Delta y_{t},\Delta z_{t}\}\right]$$ (15)

whereas for the remaining epochs, the displacement decoder network and frequency processor network were jointly optimized with the following loss function

$${\text{loss}}_{5}={\text{loss}}_{4}+\alpha_{4}\mathrm{MSE}\left\{\text{FreqNet}\left[\text{DispNet}\left(S\right)\right],\{A_{x},A_{y},A_{z}\}\right\}$$ (16)

A similar strategy was also applied for the optimization of the digital backends for the Fresnel lens array and random diffusors used for comparison to the jointly optimized diffractive vibration monitoring system.

### Experimental design and testing

We experimentally validated the feasibility of the diffractive vibration monitoring system by deploying 3D-printed diffractive layers (Stratasys, Objet30 Pro) at the first level of a four-level structure mounted on a programmable shake table. Millimeter-wave illumination at λ = 3 mm, generated by a tunable wave source (MI-WAVE, 840WF-10) with an antenna (MI-WAVE, 261 W-15) was incident to the reflective diffractive layer at 30° to the normal direction of the diffractive layer. The axial distance between the emitting antenna and the reflective diffractive layer was ~0.18 m. The reflected wave was captured by two millimeter-wave sensors (MI-WAVE, 950 W) and measured by an oscilloscope that recorded the voltage response. The center of the detectors was separated by a distance of 34 mm. Laser rangefinders (Banner Engineering, LE550DQ; sampling rate: 256 Hz) were positioned at the height of each floor and directed toward the corresponding level of the structure to measure its dynamic displacement. These measurements were used as ground truth displacements for each level. For the 1D vibration experiments, the structure was excited by various types of perturbations, including white noise, manual push and pull on the base level, as well as the first and the upper levels. For the 2D experiments, the shake table was programmed to deliver seismic excitation, simulating the structural displacements induced by, e.g., earthquakes. These programmed displacements input to the shake table included white noise, synthetic chirp signals and earthquake data from NGA-West2 (*48*) with the original intensity and 0.1× scaled-down versions.

The measured voltage signal and displacement were resampled to 50 Hz and synchronized to eliminate the difference in the measurement starting point. The measured voltage signal was first processed by an optimized 1D U-Net (*58*), which served as the displacement decoder network, DispNet, in our experiments. The downsampling path of the 1D U-Net used a series of convolution layers and max pooling to reduce temporal dimensions while increasing depth with feature channels of 16, 32, and 64. After the bottleneck layer of the 1D U-Net structure, the upsampling path used transposed convolutions, skip connections from the encoder to recover temporal details, and applied convolutional layers to refine the temporal features. The final 1x1 convolution maps the resulting features to the desired output channels (1D and 2D displacement predictions). FreqNet remained the same as before.

Experimental data for the 1D spectral measurements were generated by systematically applying six distinct perturbation types across three structural configurations (shown in Fig. 6B). Five independent measurements were acquired for each of these 18 resulting experimental conditions. For the digital backend optimization, the acquired data were partitioned into nonoverlapping training and testing subsets. The training set consisted of 80% of the measurements, whereas the remaining 20% was reserved for blind testing. The digital backend networks, DispNet and FreqNet, were trained jointly by minimizing an experimental loss function, $\mathrm{Lo}ss_{\exp}$, defined as

$$\begin{array}{cc} {\text{loss}}_{exp} & =\alpha_{5}\mathrm{MSE}\left\{\text{FreqNet}\left[\text{DispNet}\left(S\right)\right],\{A_{x},A_{z}\}\right\}\\ & +\alpha_{6}\mathrm{MSE}\left(\text{log}\left\{\text{FreqNet}\left[\text{DispNet}\left(S\right)\right]\right\},\text{log}\left(\{A_{x},A_{z}\}\right)\right)\\ & +\alpha_{7}\mathrm{MSE}\left[\text{DispNet}\left(S\right),\{\Delta x_{t},\Delta z_{t}\}\right] \end{array}$$ (17)

which included the linear and log-scale MSE of the vibration spectral inference of FreqNet as well as the MSE of the displacement inference of DispNet. $\alpha_{5},\alpha_{6}$, and $\alpha_{7}$ are hyperparameters, which were empirically set as 1, 2, and 1, respectively.

Similarly, for the 2D spectral measurements, a push on the fourth level was first applied before the programmable shake table started to follow the seismic data. The whole dataset was 80 to 20% separated into two sets (training and testing datasets) for the training of DispNet and FreqNet, which followed the same procedures and hyperparameters as the 1D case reported above.
