# Supplementary material for: Structural vibration monitoring with diffractive optical processors
Source: Sci Adv. 2026 Mar 4;12(10):eaea1712. doi: 10.1126/sciadv.aea1712 (PMC12959400; doi:10.1126/sciadv.aea1712)
Supplement: Supplementary file 1 — Figs. S1 to S8 Tables S1 to S3 [file sciadv.aea1712_sm.pdf]

Science Advances
AAAS

Supplementary Materials for

Structural vibration monitoring with diffractive optical processors

Yuntian Wang *et al.*

Corresponding author: Aydogan Ozcan, ozcan@ucla.edu

Sci. Adv. **12**, eaea1712 (2026)
DOI: 10.1126/sciadv.aea1712

This PDF file includes:

Figs. S1 to S8
Tables S1 to S3

Supplementary Figures

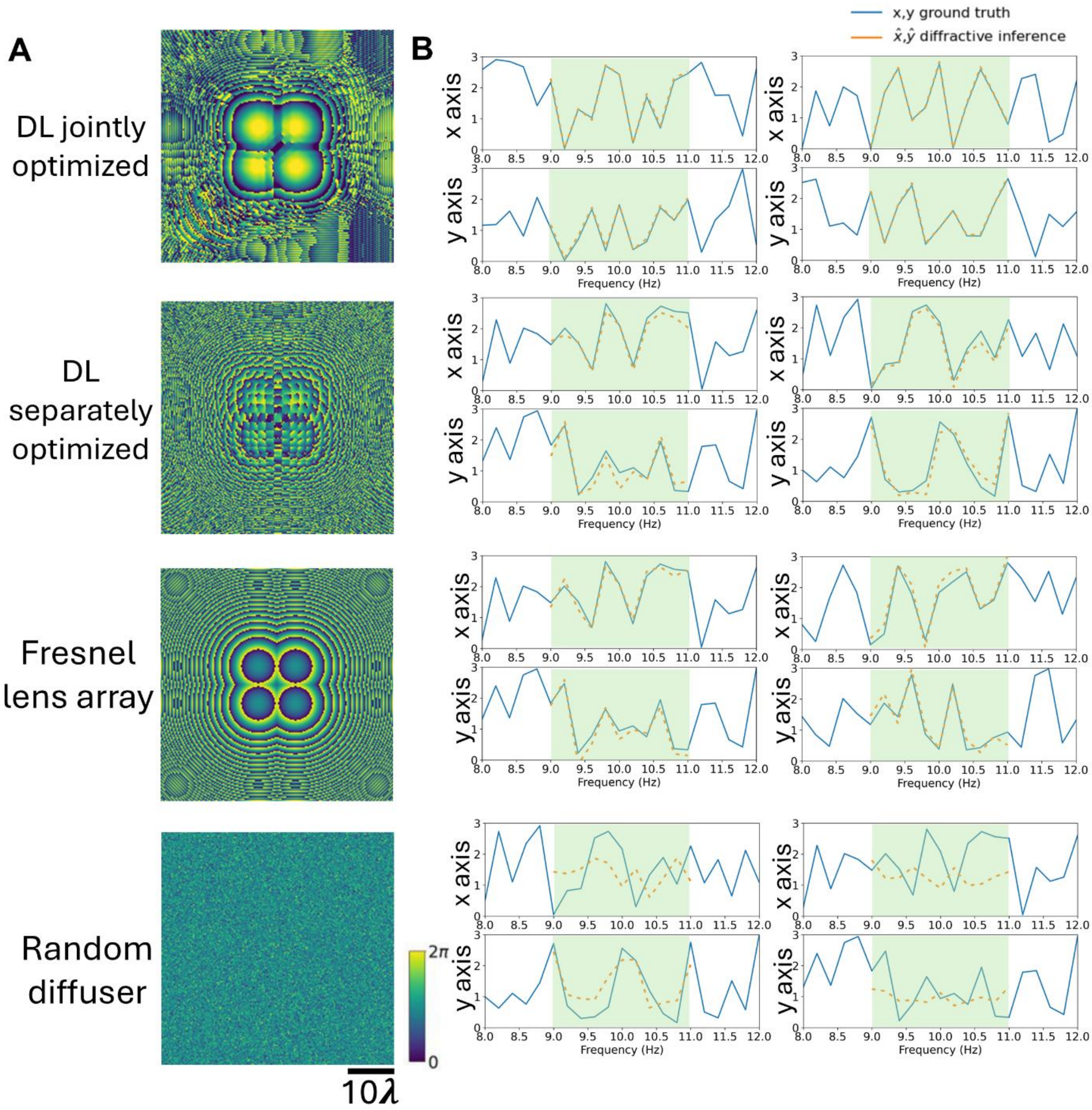

Supplementary Fig. S1. Comparison of 2D oscillation spectra inference performance across different optical configurations. (**A**) Phase modulation patterns of a jointly trained diffractive layer, a separately trained diffractive layer, a Fresnel lens array and a random phase diffuser, displayed from top to bottom, respectively. (**B**) Ground truth 2D oscillation spectra (two examples in each direction) and the diffractive inference results for each configuration. The green-shaded region highlights the frequency band of interest (9 -11 Hz), i.e., the training range of the spatial oscillations. The spectral MSE values corresponding to the

phase modulation patterns reported in the first column of **Supplementary Table 1** are 7.656×10^{-3} (jointly optimized DL), 6.507×10^{-2} (separately optimized DL), 2.826×10^{-2} (Fresnel lens array), 7.134×10^{-1} (random diffuser); $N_D = 2.96k$. The jointly optimized diffractive layer consistently achieved the lowest spectral MSE, with at least an order of magnitude improvement over other configurations – all of which used $N_D = 2.96k$ at the digital backend.

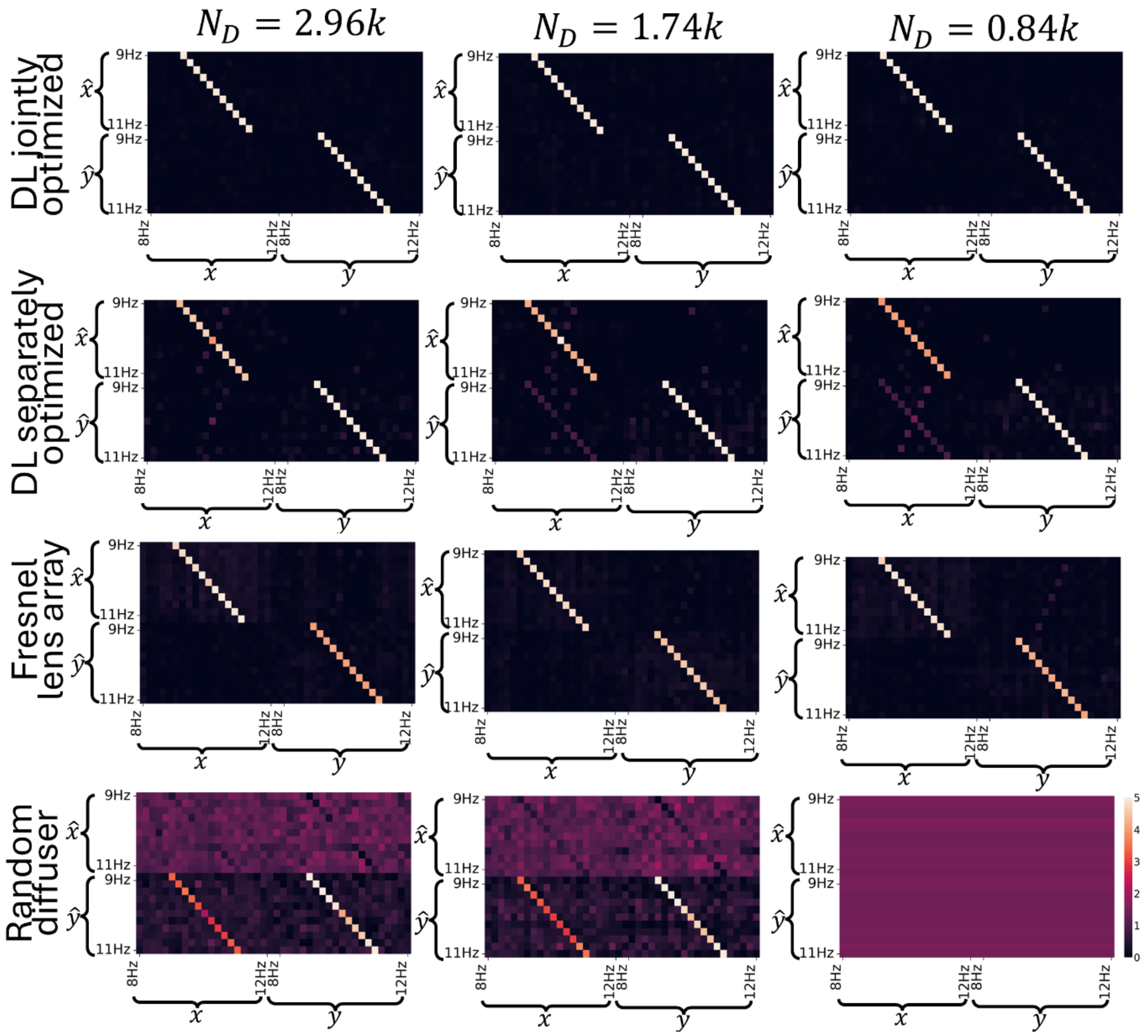

Supplementary Fig. S2. Analysis of 2D spectral inference performance. Confusion matrices for single frequency inference across various system configurations and displacement decoder sizes (N_D). The horizontal axis represents input frequencies, and the vertical axis represents the inference spectra (9-11Hz) for structural oscillations in x and y. The color bar shows the inference intensity.

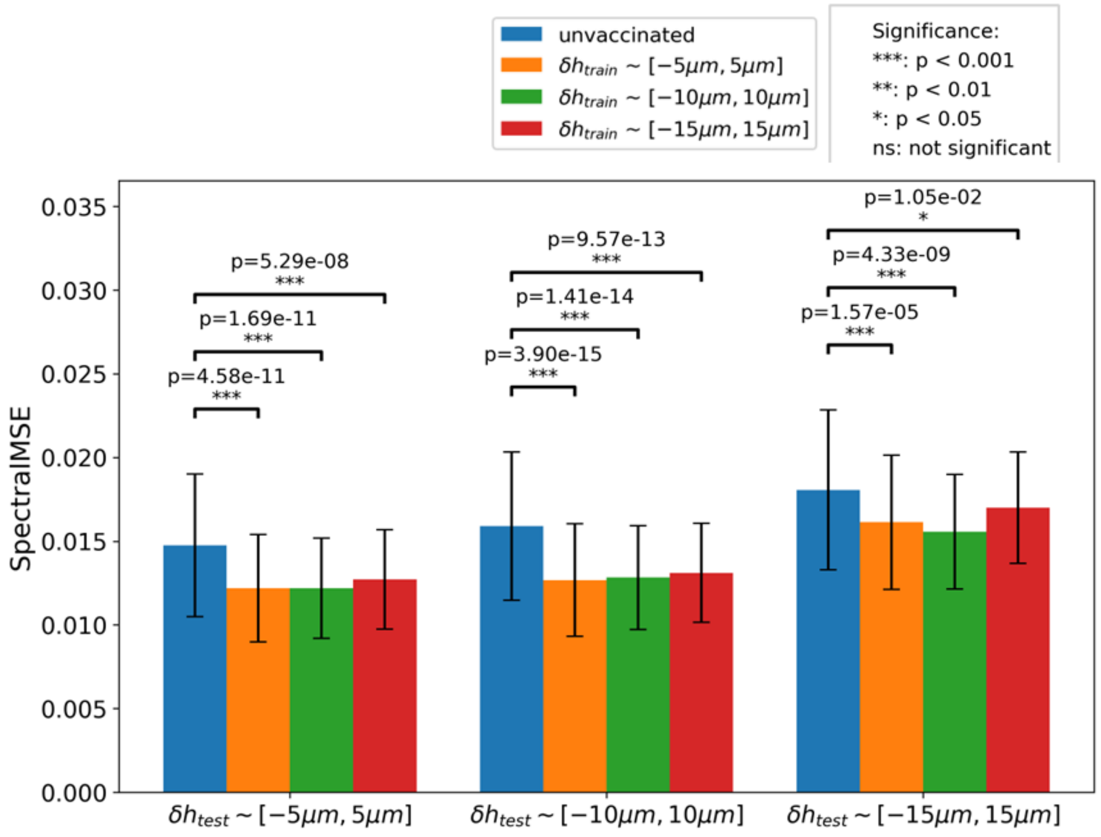

Supplementary Fig. S3. Evaluation of the diffractive vibration monitoring system under randomly introduced imperfections. The spectral MSE is reported for varying levels of random height error (δh_{test}) independently applied at each pixel of the diffractive surface during its blind testing to simulate 3D fabrication inaccuracies or other random perturbations. The performance of the standard "unvaccinated" diffractive model is compared against the "vaccinated" models, which were trained by explicitly incorporating random height noise (δh_{train}) into the diffractive layer during the joint optimization process to enhance its robustness. The error bars represent the standard deviation of the spectral reconstruction performance. Also see **Table S2** for the statistical significance of the observed differences. Asterisks denote statistical significance levels: * $p < 0.05$, ** $p < 0.01$, *** $p < 0.001$; ns: not significant.

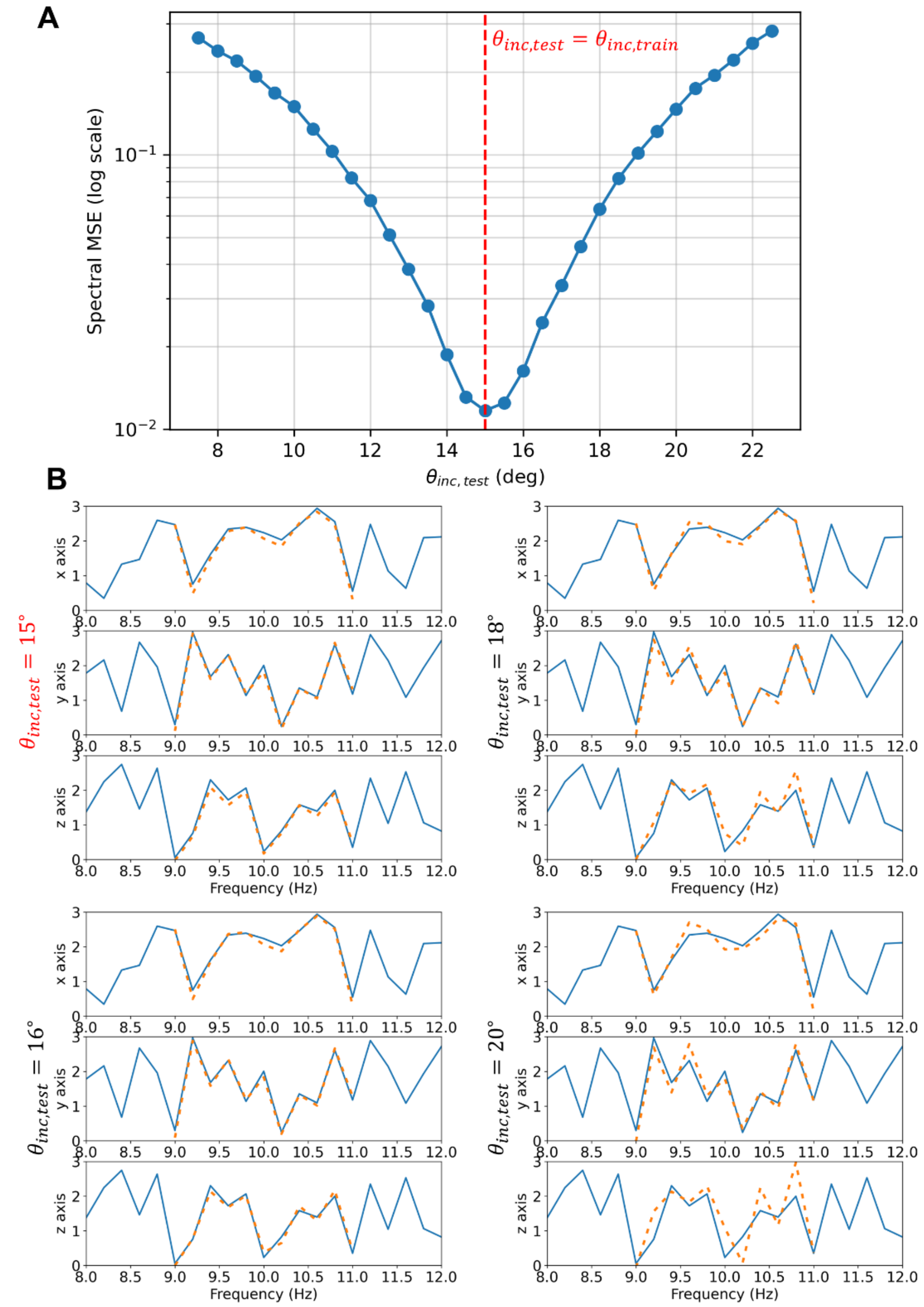

Supplementary Fig. S4. Performance analysis of the diffractive vibration monitoring system as a function of the angular misalignment of the illumination source. (**A**) The spectral MSE plotted on a logarithmic scale as a function of the incidence angle of the input wave ($\theta_{inc,test}$). The diffractive layer was optimized for an incidence angle of $\theta_{inc,train} = 15°$ (red dashed line). (**B**) Comparison of the ground truth (solid blue lines) and the reconstructed (dashed orange lines) 3D oscillation spectra for various test incidence

angles. The results demonstrate the system's angular tolerance, showing high-fidelity reconstruction near the design angle and a gradual degradation in performance as the misalignment increases.

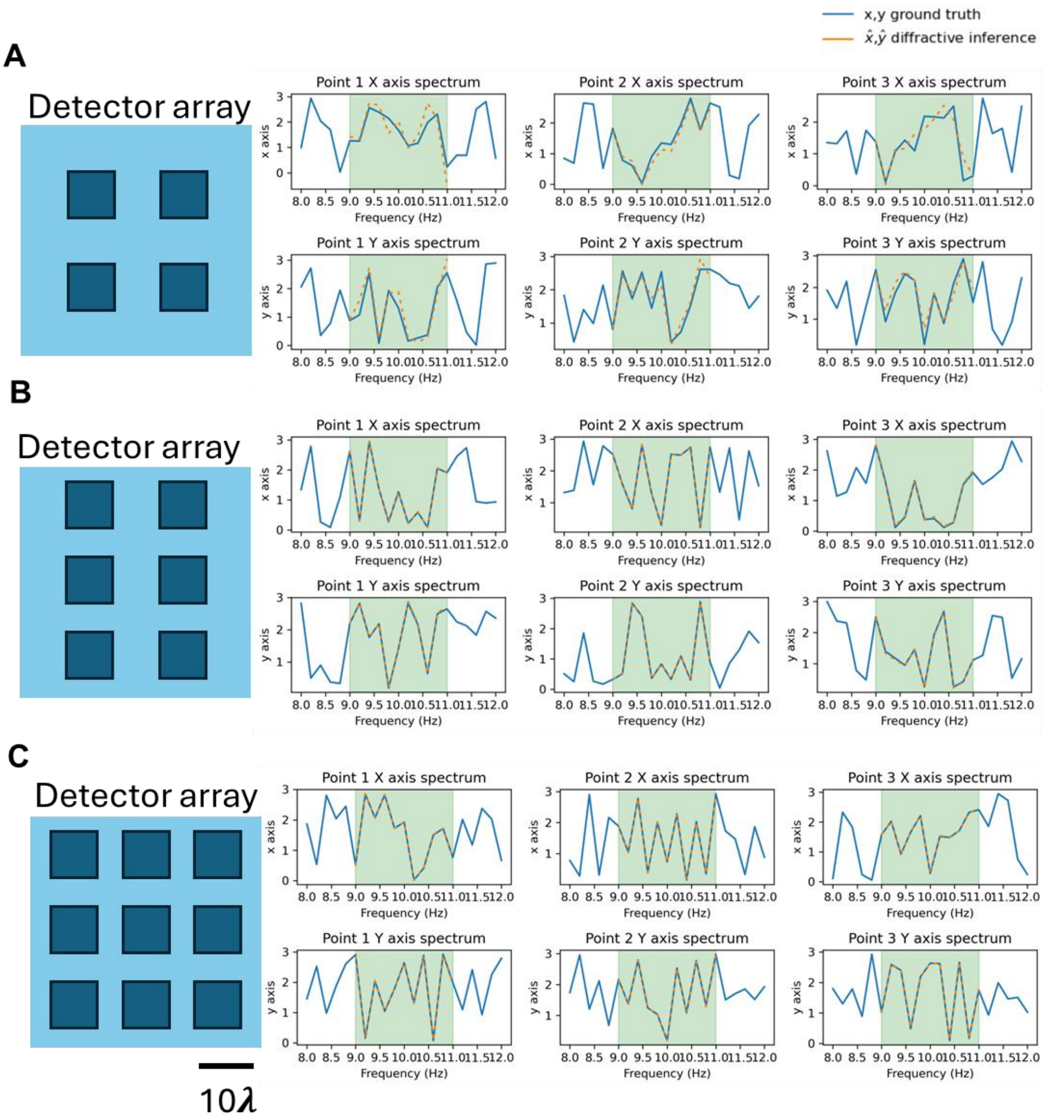

Supplementary Fig. S5. Performance comparison of simultaneous multi-point vibration monitoring with varying numbers of detectors (N_{dt}). The detector array layouts (left column) and the corresponding spectral inference results (right columns) are shown for configurations using (**A**) $N_{dt} = 4$ detectors, (**B**) $N_{dt} = 6$ detectors, and (**C**) $N_{dt} = 9$ detectors. The spectral plots compare the ground truth (solid blue lines) and the diffractive inference (dashed orange lines) for three monitored points along both the x and y axes. The under-determined system in (**A**) exhibits significantly larger inference errors ($1.446 \times 10^{-1} \pm 4.646 \times 10^{-2}$), while the determined (inference error $5.301 \times 10^{-4} \pm 7.244 \times 10^{-4}$) and over-determined systems (inference error $3.555 \times 10^{-4} \pm 2.772 \times 10^{-4}$) in (**B**) and (**C**), respectively, yield higher fidelity

reconstructions. Also see **Table S3** for the statistical significance of the observed differences.

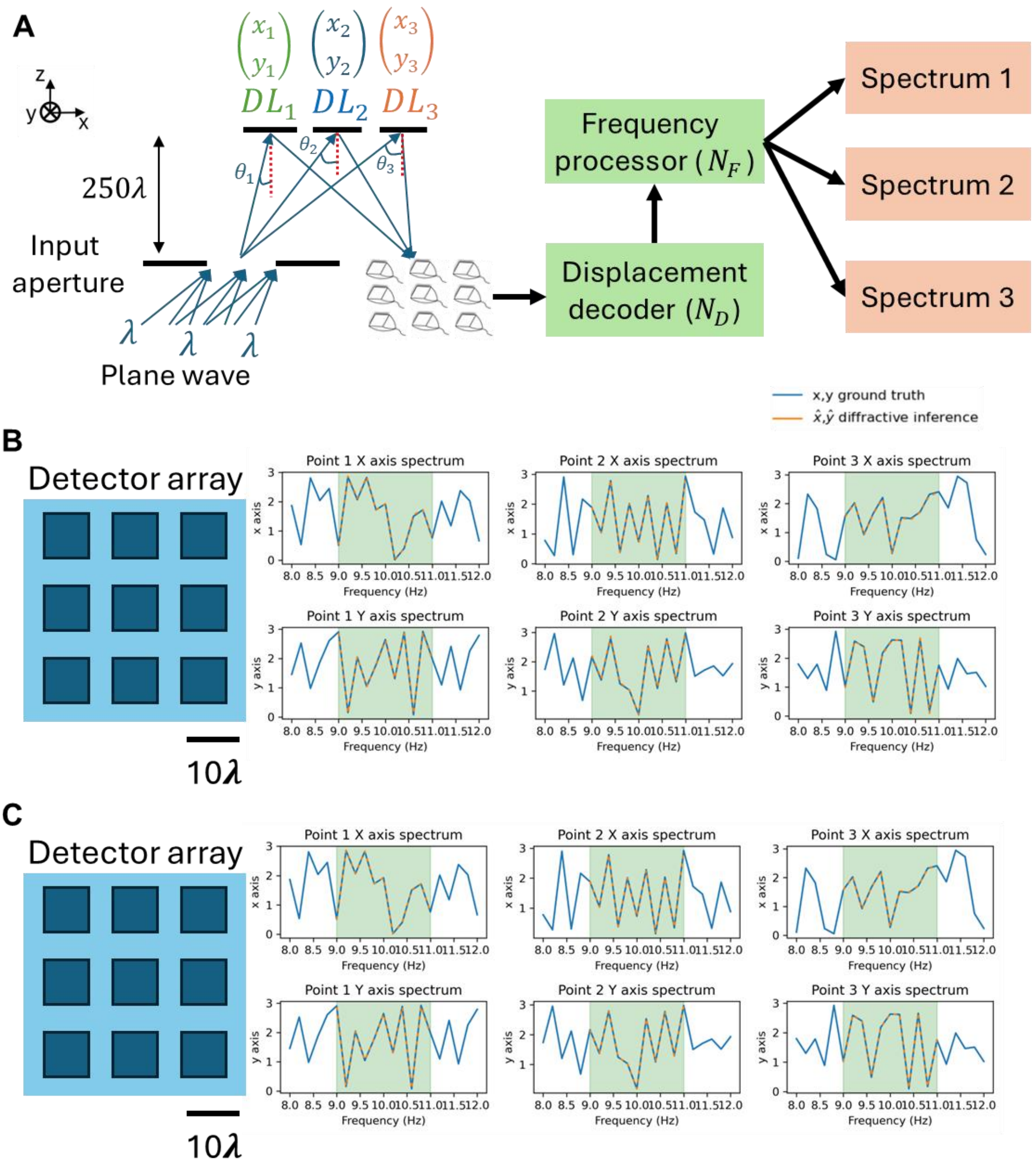

Supplementary Fig. S6. Performance comparison between monochrome and wavelength-multiplexed configurations for simultaneous multi-point vibration monitoring. (**A**) Schematic overview of the monochrome diffractive system for simultaneous multi-point vibration monitoring. (**B**) Spectral inference results for the monochrome configuration (Spectral MSE $7.175 \times 10^{-4} \pm 4.368 \times 10^{-4}$), where all three monitoring points are simultaneously illuminated by the same wavelength (λ) and measured/monitored by a

9-pixel detector array, (i.e., $N_{dt} = 9$). (**C**) Spectral inference results for the wavelength-multiplexed configuration (as described in Fig.5 of the main text). Utilizing the same detector array geometry ($N_{dt} = 9$), the wavelength-multiplexed system demonstrates superior spectral reconstruction fidelity (spectral MSE $3.555 \times 10^{-4} \pm 2.772 \times 10^{-4}$ with a p value of 2.196×10^{-10}) compared to the monochrome approach.

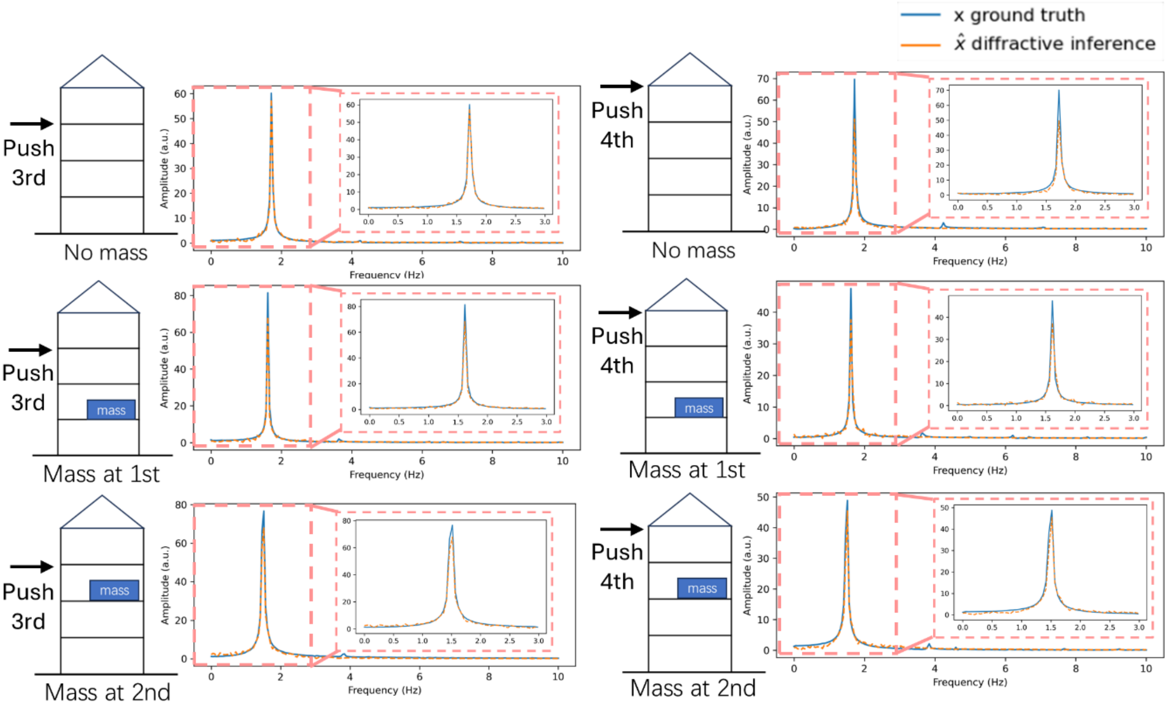

Supplementary Fig. S7. Additional experimental results of the 1D diffractive vibration monitoring system using a millimeter-wave source. Spectral inference results of different configurations with various types of perturbations and structures are compared against the ground truth.

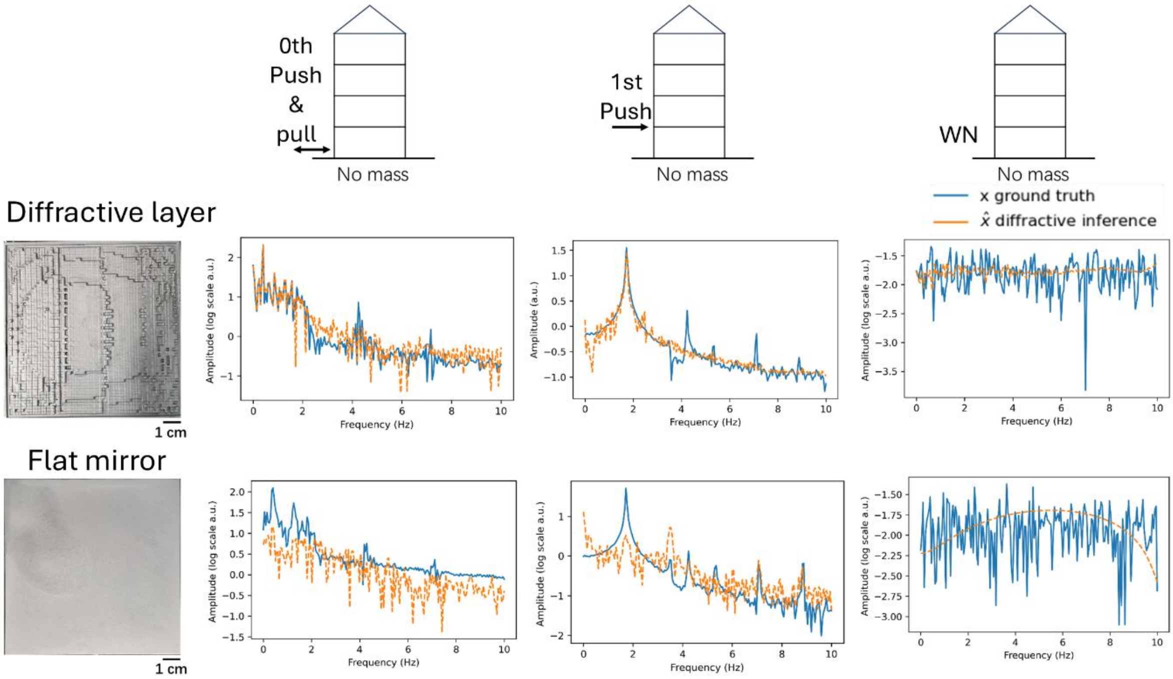

Supplementary Fig. S8. Experimental performance comparison of the 1D diffractive vibration monitoring system between the optimized diffractive layer and a reflective flat mirror that are both 3D printed. The non-zero energy at zero frequency was caused by the displacement of the base level (0th level) due to the manual perturbation. WN: white noise.

**Spectral MSE**	$N_D = 2.96k$	$N_D = 1.74k$	$N_D = 0.84k$
Diffractive layer (jointly optimized)	7.656×10^{-3}	1.143×10^{-2}	1.269×10^{-2}
Diffractive layer (separately optimized)	6.507×10^{-2}	7.702×10^{-2}	9.195×10^{-2}
Fresnel lens array	2.826×10^{-2}	4.179×10^{-2}	4.864×10^{-2}
Random diffuser	7.134×10^{-1}	7.218×10^{-1}	7.507×10^{-1}

Supplementary Table 1. 2D spectral MSE results of different optical configurations, evaluated as a function of the number of trainable parameters (N_D) of the displacement decoder network.

**P value**	δh_{test}~$[-5\mu m, 5\mu m]$	δh_{test}~$[-10\mu m, 10\mu m]$	δh_{test}~$[-15\mu m, 15\mu m]$
δh_{train}~$[-5\mu m, 5\mu m]$	4.584×10^{-11}	3.901×10^{-15}	1.565×10^{-5}
δh_{train}~$[-10\mu m, 10\mu m]$	1.693×10^{-11}	1.411×10^{-14}	4.332×10^{-9}
δh_{train}~$[-15\mu m, 15\mu m]$	5.286×10^{-8}	9.583×10^{-13}	1.045×10^{-2}

Supplementary Table 2. Statistical significance of the performance improvements achieved by the vaccination strategy. P-values calculated between the spectral MSE distributions of the baseline (unvaccinated) models and the models vaccinated with varying amplitudes of uniformly distributed training noise (δh_{train}). These statistical comparisons were conducted using 100 independent tests across different testing noise conditions (δh_{test}), confirming that the performance differences observed between the unvaccinated baseline models and each of the vaccinated models are statistically significant, in favor of the vaccinated diffractive designs.

	$N_{dt} = 4$	$N_{dt} = 6$	$N_{dt} = 9$
**Spectral MSE** **($\mu \pm \sigma$)**	1.446×10^{-1} $\pm 4.646 \times 10^{-2}$	5.301×10^{-4} $\pm 7.244 \times 10^{-4}$	3.555×10^{-4} $\pm 2.772 \times 10^{-4}$

$p = 1.37 \times 10^{-52}$
***

$p = 3.73 \times 10^{-2}$
*

$p = 1.26 \times 10^{-52}$
***

Supplementary Table 3. Spectral MSE results and the statistical significance of wavelength multiplexed multi-point monitoring performance improvement with increasing number of detectors (N_{dt}). The spectral MSE is calculated by 100 samples and reported with mean (μ) and standard deviation (σ). The statistical significance of the spectral MSE improvement between different N_{dt} designs is also reported. Asterisks denote significance levels: * $p < 0.05$, ** $p < 0.01$, *** $p < 0.001$.
